# The Composite 259-kb Plasmid of *Martelella mediterranea* DSM 17316^T^–A Natural Replicon with Functional RepABC Modules from *Rhodobacteraceae* and *Rhizobiaceae*

**DOI:** 10.3389/fmicb.2017.01787

**Published:** 2017-09-21

**Authors:** Pascal Bartling, Henner Brinkmann, Boyke Bunk, Jörg Overmann, Markus Göker, Jörn Petersen

**Affiliations:** Leibniz-Institute DSMZ–German Collection of Microorganisms and Cell Cultures Braunschweig, Germany

**Keywords:** RepABC-type plasmids, compatibility, type IV secretion systems, plasmid fusion, comparative genomics, horizontal gene transfer

## Abstract

A multipartite genome organization with a chromosome and many extrachromosomal replicons (ECRs) is characteristic for *Alphaproteobacteria*. The best investigated ECRs of terrestrial rhizobia are the symbiotic plasmids for legume root nodulation and the tumor-inducing (Ti) plasmid of *Agrobacterium tumefaciens*. RepABC plasmids represent the most abundant alphaproteobacterial replicon type. The currently known homologous replication modules of rhizobia and *Rhodobacteraceae* are phylogenetically distinct. In this study, we surveyed type-strain genomes from the One Thousand Microbial Genomes (KMG-I) project and identified a roseobacter-specific RepABC-type operon in the draft genome of the marine rhizobium *Martelella mediterranea* DSM 17316^T^. PacBio genome sequencing demonstrated the presence of three circular ECRs with sizes of 593, 259, and 170-kb. The rhodobacteral RepABC module is located together with a rhizobial equivalent on the intermediate sized plasmid pMM259, which likely originated in the fusion of a pre-existing rhizobial ECR with a conjugated roseobacter plasmid. Further evidence for horizontal gene transfer (HGT) is given by the presence of a roseobacter-specific type IV secretion system on the 259-kb plasmid and the rhodobacteracean origin of 62% of the genes on this plasmid. Functionality tests documented that the genuine rhizobial RepABC module from the *Martelella* 259-kb plasmid is only maintained in *A. tumefaciens* C58 (*Rhizobiaceae*) but not in *Phaeobacter inhibens* DSM 17395 (*Rhodobacteraceae*). Unexpectedly, the roseobacter-like replication system is functional and stably maintained in both host strains, thus providing evidence for a broader host range than previously proposed. In conclusion, pMM259 is the first example of a natural plasmid that likely mediates genetic exchange between roseobacters and rhizobia.

## Introduction

RepABC-type plasmids play a crucial role for the multipartite genome organization and the lifestyle of rhizobia (Pappas and Cevallos, 2011). Long-known examples are the pathogenic tumor-inducing (Ti) plasmid of *Agrobacterium tumefaciens* and the symbiotic nodulation (pSym) plasmids of the genus *Rhizobium*. RepABC-type plasmids comprise up to 50% of the rhizobial genome and represent the by far most abundant replicon type of these soil bacteria (Pappas and Cevallos, 2011). *Rhizobium etli* CFN42 harbors eight RepABC operons that are located on six extrachromosomal replicons (ECRs; González et al., 2006). The relevance of ECRs for the marine roseobacter group (*Rhodobacterceae*) is exemplified by photosynthesis, flagellar and biofilm plasmids (Petersen et al., 2012; Michael et al., 2016). Roseobacters contains at least four different plasmid types (RepA, RepB, DnaA-like, RepABC) with more than 20 compatibility groups (Petersen, 2011). Nine different compatibility groups of RepABC-type plasmids, which can stably coexist in the same cell, have been identified in this lineage (Petersen et al., 2009).

RepABC modules are specific for *Alphaproteobacteria* and contain three genes, the *repA* and *repB* partitioning genes as well as the replication gene *repC*, which are arranged in a characteristic operon (Pinto et al., 2012). The structure of RepABC type plasmids coincides with the localization of the origin of replication (*ori*) within the protein coding part of the replicase (*repC*) and the presence of a regulatory antisense RNA between *repB* and *repC* (Weaver, 2007). Conserved palindromes of RepABC-type plasmids that serve as cis-acting anchors for RepB proteins are indispensable for the successful partitioning of these low copy number replicons. The RepA/RepB system of RepABC-type plasmids is homologous to the universal ParA/ParB partitioning system of the bacterial chromosome and other tripartite plasmids (Petersen et al., 2011).

Horizontal gene transfer (HGT) correlates with major evolutionary transitions (Nelson-Sathi et al., 2015) and the process is *inter alia* mediated by phages and conjugative plasmids. Plasmid mobilization is ensured by type IV secretion systems (T4SS), representing a conserved export apparatus that is also used by several pathogenic bacteria for DNA and protein secretion (Cascales and Christie, 2003). The role of plasmid conjugation for the rapid adaptability of bacteria is exemplified by the emergence of multi-resistant hospital strains as a consequence of massive antibiotic (mis) use in medicine and livestock husbandry (Palmer et al., 2010). Many extrachromosomal elements are adapted to their host and do not stably maintain in distantly related bacteria (narrow-host-range plasmid; Kües and Stahl, 1989). A prominent example is the Ti plasmid from *A. tumefaciens* C58 that can be conjugated into *Escherichia coli* but requires an additional replication system for stable maintenance in *Enterobacteriaceae* (*Gammaproteobacteria*; Holsters et al., 1978). Naturally occurring broad-host-range vectors from *E. coli* were genetically engineered and serve as crucial tools for molecular biology (see e.g., Kovach et al., 1995).

Conjugative plasmid transfer is an important factor in the evolution of rhizobia (Ding and Hynes, 2009; López-Guerrero et al., 2012) and has been proposed as the major driving force for the rapid adaptation of roseobacters to novel ecological niches (Petersen et al., 2013). In nitrogen-fixing rhizobia, symbiotic plasmids mediate legume root nodulation and define the particular plant host (Perret et al., 2000; Gibson et al., 2008). The pSym plasmid has been horizontally exchanged between different sympatric *Rhizobium* species, thereby transferring the capacity to nodulate the same host cell, i.e., the common bean *Phaseolus vulgaris* (Pérez Carrascal et al., 2016). Comparative genome analyses of *Rhodobacteraceae* showed that T4S systems for plasmid mobilization are typically located on RepABC plasmids, and experimental conjugation of natural plasmids has recently become feasible (Patzelt et al., 2016). The wealth of more than 400 draft genomes allowed tracing natural plasmid transfer among genus barriers in the roseobacter group (Petersen and Wagner-Döbler, 2017). However, the recombination rate between bacteria exponentially drops with increasing sequence divergence (Fraser et al., 2007), which depends, e.g., on the limited host range of mobilizable plasmids.

One purpose of the current study was the experimental validation of our *in silico* prediction that RepABC-type plasmids can not be transferred stably between *Rhizobiaceae* and *Rhodobacteraceae*. They are equivalent in structure and function but can clearly be distinguished by phylogenetic analyses (Petersen et al., 2009). Eight RepABC compatibility groups from roseobacters (*Rhodobacteraceae*) have a common origin and were once recruited from a rhizobial donor, but the respective plasmids have not been identified in rhizobia. The strict phylogenetic separation of these plasmid modules allows for a reliable genomic differentiation between both alphaproteobacterial orders, thus providing indirect evidence for functional constraints resulting in a narrow-host-range. However, the supposed ecological separation between the soil and the ocean, which would limit the physical contact for conjugation, is less pronounced than *a priori* assumed. Several rhizobial lineages, such as the genus *Martelella* are adapted to saline habitats (Rivas et al., 2005) and roseobacters represent a paraphyletic group associated with non-marine *Rhodobacteraceae* including the genus *Paracoccus* (Simon et al., 2017).

In the current study we experimentally document that rhizobial RepABC plasmids do not replicate in *Phaeobacter inhibens* DSM 17395 (*Rhodobacteraceae*), but we provide the first example of a natural plasmid that can be stably maintained in both rhizobia and roseobacters. This composite plasmid from the marine rhizobium *Martelella mediterranea* DSM 17316^T^ originated from a plasmid fusion and still harbors rhizobial and rhodobacteral RepABC cassettes thus overcoming the limits of their host range.

## Results and discussion

### Identification of a *Rhodobacteraceae*-specific RepABC plasmid replication module in *Martelella mediterranea* DSM 17316^T^

Extensive data mining in roseobacters (*Rhodobacteraceae*) was the basis for the detection of nine compatibility groups of RepABC plasmids (Petersen et al., 2009), and recent studies indicated that this replicon type is crucial for HGT via conjugation (Petersen et al., 2013; Frank et al., 2015; Patzelt et al., 2016). The discoveries of the current study benefit from the Genomic Encyclopedia of Bacteria and Archaea (GEBA) genome sequencing project, which was aimed to fill phylogenetic gaps in the tree of life (Wu et al., 2009; Mukherjee et al., 2017), and the follow-up study of one thousand microbial genomes (KMG-I) that was focused on type strains (Kyrpides et al., 2014). More than a third of the selected strains from the latter project were *Proteobacteria* with 107 alphaproteobacterial representatives and 17 *Rhodobacteraceae* that were used to improve our reference data set of plasmid modules. However, we also investigated the distribution of RepABC operons in 35 novel rhizobial genomes, because this order previously served as a distantly related natural outgroup for *Rhodobacteraceae*-specific ECRs (Petersen et al., 2009). BLASTP searches with the RepC-2 replicase of the 126-kb RepABC plasmid from *Dinoroseobacter shibae* DFL12^T^ (pDSHI03; WP_012187065.1) allowed us to identify typical rhizobial homologs with a moderate protein identity of up to 40%, which is exemplified by the Ti-plasmid of *A. tumefaciens* C58 (36% identity). The sole and striking exception is a highly conserved RepABC module from the marine rhizobium *M. mediterranea* DSM 17316^T^ (Rivas et al., 2005), whose replicase exhibits a conspicuous conservation of 58% identity. This finding was independently validated by analogous BLASTP searches with the adjacent RepA and RepB partitioning proteins, thereby documenting that *Martelella*'s RepABC module is indistinguishable from genuine operons of *Rhodobacteraceae*.

### Genome sequencing of *Martelella mediterranea* DSM 17316^T^

#### Establishment of a finished genome with the PacBio technique

We identified the roseobacter-specific RepABC plasmid replication operon of *Martelella* on a linear 105-kb DNA fragment (scaffold_16.17, NZ_AQWH01000017), which has been established in the first phase of the type strain sequencing project One Thousand Microbial Genomes (KMG-I; Kyrpides et al., 2014). The scaffold also contains a complete type IV secretion system (T4SS; Cascales and Christie, 2003) and a characteristic post-segregational killing system (PSK) encoding a stable toxin and an unstable antitoxin (Zielenkiewicz and Cegłowski, 2001) thus indicating that it might still represent a functional plasmid. However, meaningful analyses of extrachromosomal elements and the systematic investigation of HGT via conjugation essentially depend on a complete genome sequence without any gaps and uncertain contig-affiliations. Accordingly, the genome of *M. mediterranea* DSM 17316^T^ was sequenced with our PacBio platform. Based on a 130-fold sequence coverage and subsequent Illumina correction, we obtained a finished genome of highest quality with a size of 5.7 Mb, harboring four circular replicons representing the 4.7 Mb chromosome and three large RepABC-type ECRs with sizes of 593-, 259-, and 170-kb (Figure S1; CP020330 to CP020333). Four scaffolds including the 105-kb fragment perfectly match with the 259-kb plasmid pMM259 thus providing independent evidence for the technical reliability of both sequencing approaches, i.e., the initial Illumina assembly and our newly established PacBio genome. Hereby, tandem repeats of transposase and recombinase genes flanking the Illumina scaffolds are limiting *de novo* genome assembly approaches based on short reads. In return the newly established complete *Martelella* genome exemplifies the repeat-resolving power of long reads established by PacBio sequencing (Bleidorn, 2016).

#### Gene content of the extrachromosomal elements

The 170-kb replicon pMM170 contains many sugar transport systems and the 593-kb ECR pMM593 holds a striking amount of TRAP- and ABC-transporters (Davidson et al., 2008; Fischer et al., 2010). Accordingly, both extrachromosomal elements might have an important function for *Martelella*'s metabolite exchange with the environment. The composite plasmid pMM259, which harbors the rhodobacteral RepABC module of interest and a rhizobial equivalent (Figure S1), contains two operons for copper export (Cu^+^; Mame_05011-05014, Mame_05047, Mame_05054), two operons for cadmium or zinc export (Mame_04956-04962, Mame_04977-04982) and an arsenate-resistance cassette (Mame_05000-05003), indicating that it represents a resistance plasmid for the detoxification of heavy metals. A specific exposure to toxic metal-ions has not been reported for the natural habitat of *M. mediterranea*, the subterranean Lake Martel on Mallorca (Rivas et al., 2005), but microorganisms are very sensitive to long time exposures of even moderate concentrations of heavy metals (Gadd and Griffiths, 1978).

### Phylogenetic analysis of *Martelella*'s *Rhodobacteraceae*-specific plasmid RepABC operon

#### Global phylogeny of RepC-type replicases

**Figure 1:**
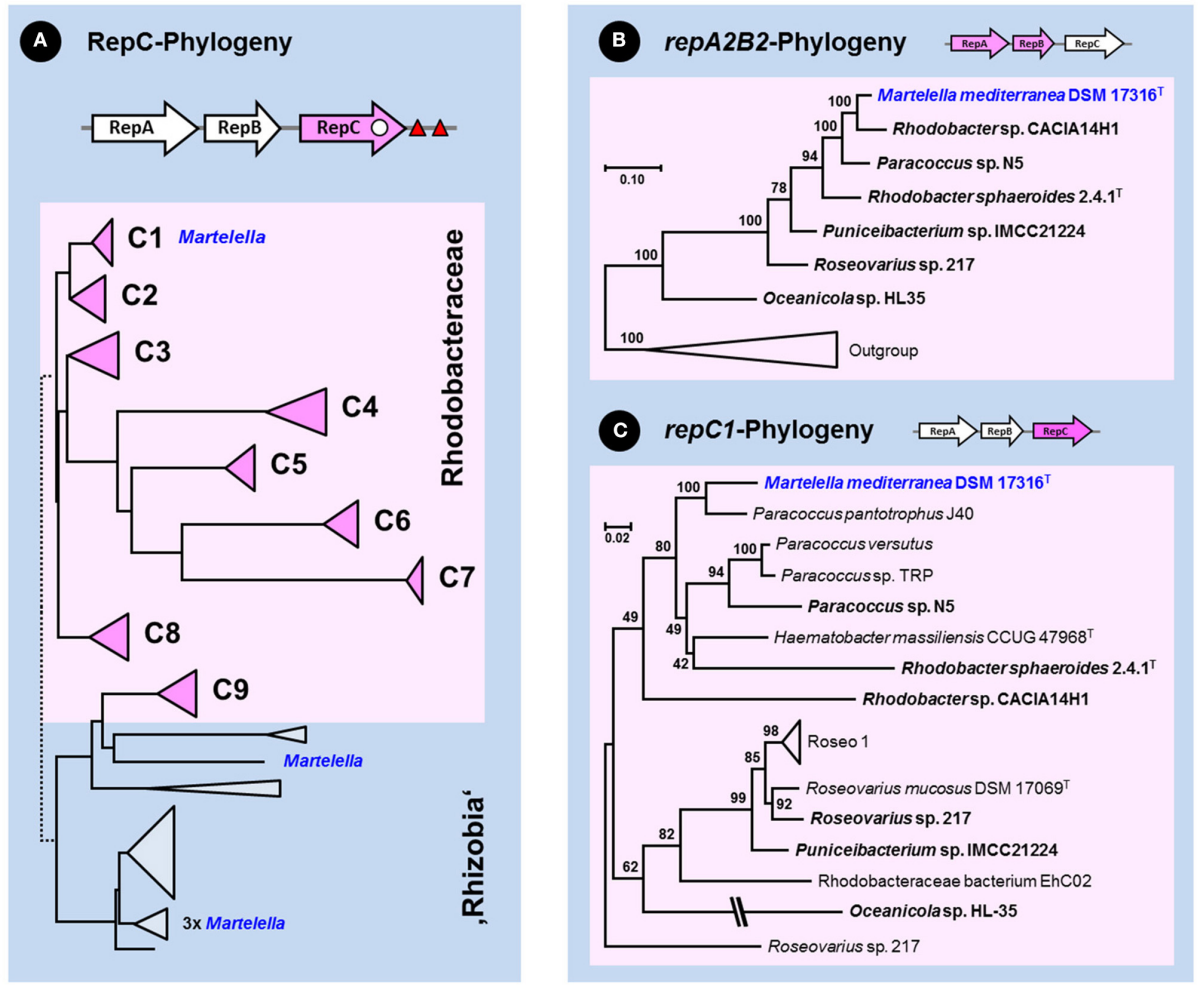
Phylogenetic analyses of the RepABC-type plasmid replication modules from *Rhodobacteraceae*. The pink color of the schematic RepABC operon above the respective phylogenetic tree indicates the analyzed gene. The origin of replication (*ori*) and conserved palindromes for plasmid partitioning are indicated by a white dot and red triangles, respectively. The localization of rhizobial RepC sequences from *Martelella mediterranea* DSM 17316^T^ is highlighted in blue. **(A)** Schematic Neighbor Joining tree of 121 RepC replicase protein sequences from *Rhodobacteraceae* and rhizobia (see Figure S2). Subtrees of the nine compatibility groups C1–C9 from roseobacters are shown by pink triangles and rhodobacteracean subtrees are highlighted by a rose box. **(B,C)** Subanalysis of nucleotide sequences from concatenated *repA2B2* genes and the *repC1* gene. *Rhodobacteraceae* with a reshuffled RepA2B2A1 replication modules are highlighted in bold (Figures S6, S7). The final taxon sampling for the localization of *M. mediterranea* was determined by comprehensive analyses of RepA, RepB and RepC proteins belonging to compatibility groups 1 and 2 (Figures S3–S5).

The genome of *M. mediterranea* harbors four RepABC replication modules [pMM593 (Mame_04351-04353, Mame_04880-04882), pMM259 (Mame_04944-04946), pMM169 (Mame_05119-05121)] and a solitary replicase gene [pMM259 (Mame_04960)]. The phylogenetic position of the respective RepC proteins was determined based on a set of roseobacterial and mostly rhizobial reference sequences largely corresponding to those of our former study (Petersen et al., 2009), which allowed us to differentiate between the nine different compatibility groups from *Rhodobacteraceae* originating from two ancient acquisitions (I: C1 to C8, II: C9). The phylogenetic tree, which was calculated based on 121 RepC replicases, showed that four *Martelella* sequences are located in the rhizobial part of the tree (blue color; Figure 1A, Figure S2). In contrast, the newly identified RepC protein of interest is placed amidst other rhodobacteral sequences in the distinct subtree C1 statistically supported by a 100% bootstrap proportion (BP; rose color; Figure S2). The internal branching pattern of the distinct subtree C1 is only poorly resolved due to the phylogenetically broad sequence sampling including the extremely divergent subtrees C4–C7, which resulted in only 145 comparable amino acid (aa) alignment positions (Figure S2). Accordingly, further analyses were focused on the *Rhodobacteraceae*-specific subtrees C1 and C2, whose sister-group relationship is solidly supported (85% BP; Figure 1, Figure S2). We systematically searched the public sequence databases and investigated 81 different complete RepABC operons of the compatibility groups 1 and 2 (Petersen et al., 2009). Comprehensive phylogenetic subanalyses of all three genes were performed in order to detect the closest relative(s) of the roseobacterial RepABC module from *M. mediterranea*.

#### Phylogenetic analyses of partitioning proteins (RepA, RepB) and the replicase RepC

Our subanalyses of proteins from RepABC modules belonging to the compatibility groups -1 and -2 resulted in a major improvement of the phylogenetic resolution with increased bootstrap support (Figures S3–S5). The RepA and RepB trees have comparable branching patterns, which mirrors their concerted evolution in a functional partitioning operon, and the best resolution was obtained in the RepB analysis (Figure S4). Both partitioning proteins of *Martelella* (Mame_04880-04881) are located in subtrees A2 and B2 (green sequences, 100% BP), which is *a priori* surprising because they show a deviating localization compared to the replicase RepC that is located in subtree C1 (Figures S2, S5). Our previous study showed a synchronous evolution of all three genes of the RepABC operon (Petersen et al., 2009), which is here exemplified by *Dinoroseobacter shibae* DFL12^T^, *Pseudooceanicola batsensis* HTCC 2597^T^, *Roseovarius indicus* B108^T^ and *R. atlanticus* R12B^T^, four roseobacter type-strains that harbor RepABC operons of both compatibility groups (A1B1C1, A2B2C2; Figures S3–S5). Recombination events between a partitioning module of one compatibility group with a replicase of another compatibility group are rare, but they have previously been reported for the A2B2C1 module from *Roseovarius* sp. 217 (Petersen et al., 2009). The respective partitioning proteins of *M. mediterranea* (RepA, RepB) group together with *Roseovarius* sp. 217 and five other *Rhodobacteraceae* in a distinct branch of subtree -2 (highlighted in green, 100% BP; Figures S3, S4), in contrast to their replicases (RepC) that are all located in subtree -1, which reflects the common origin of the reshuffled A2B2C1 module.

#### Detection of RepABC-1 and -2 specific palindromes

An independent criterion for the classification of RepABC-type plasmid replication modules is the presence of specific palindromes that also allow for differentiating between the nine compatibility groups in *Rhodobacteraceae* (Petersen et al., 2009). The highly conserved inverted repeats with a length of 14 nucleotides are in RepABC-1 and -2 modules typically located in close proximity of the RepABC operon downstream of RepC (Figure 1). Accordingly, we investigated the sequences of 36 RepABC modules starting 500 base pairs (bp) upstream of the RepA start codon and ending 500 bp downstream of the RepC stop codon. The sampling was focused on the A2B2C1 module of *M. mediterranea* DSM 17316^T^ and our model organism *D. shibae* DFL12^T^, which served as a reference due to the presence of two plasmids with characteristic A1B1C1 and A2B2C2 modules (see above; Petersen et al., 2013). All but three of the investigated plasmid replication systems contain two adjacent copies of the specific palindrome separated by only 11 to 42 bp (Table 1). Necessities of partitioning appeared to result in a nearly universal conservation of the palindrome motifs TTAACAG/CTGTTAA for compatibility group -1 and TTCACAG/CTGTGAA for compatibility group -2. A single reciprocal nucleotide exchange in the third and third to last palindrome position (A:C, T:G) is responsible for plasmid compatibility and thus the stable co-existence e.g., of the 86-kb RepABC-1 and the 126-kb RepABC-2 replicons in *D. shibae* (Wagner-Döbler et al., 2010). *Martelella* and five additional *Rhodobacteraceae* with A2B2C1 modules harbor the characteristic doublet of compatibility group -2 palindromes, which is indistinguishable from those of genuine A2B2C2 plasmids. The phylogenetic localization of their partitioning genes in subtrees A2 and B2 reflects, in combination with the presence of compatibility group -2 palindromes, a case example of co-evolution based on functional constraints (Figures S3, S4; Table 1). The palindrome represents the highly specific cis-acting DNA recognition site for the partitioning protein ParB, whose smooth interaction is the prerequisite for successful plasmid distribution during bacterial cell division (Pinto et al., 2012). The lack of conserved palindromes in *Oceanicola* sp. HL-35, the sixth strain with an A2B2C1 operon (Figures S3–S5), might reflect the inactivation of its RepABC module. This prediction is supported by the early-branching position of strain HL-35 in the RepC tree (Figure S5) that could represent a phylogenetic long-branch attraction artifact (LBA, Philippe et al., 2005). In contrast, our phylogenetic analyses showed neither a conspicuous position nor prolonged branches for *M. mediterranea*'s RepA, RepB and RepC sequences (Figures S3–S5), thus indicating that its A2B2C1 plasmid module should be still functional at least in roseobacters (*Rhodobacteraceae*). Further phylogenies clearly documented a common origin of the *repA2B2* partitioning operon together with *Rhodobacter* sp. CACIA14H1 and of the *repC1* gene together with *Paracoccus pantotrophus* J40 (Figures 1B,C; Supplemental Material S1). This distribution reflects the frequent reshuffling of the replicase in A2B2C1 modules and moreover showed that the genuine rhodobacteracean donor of *Martelella*'s RepABC cassette has not been detected yet.

**Table 1:**
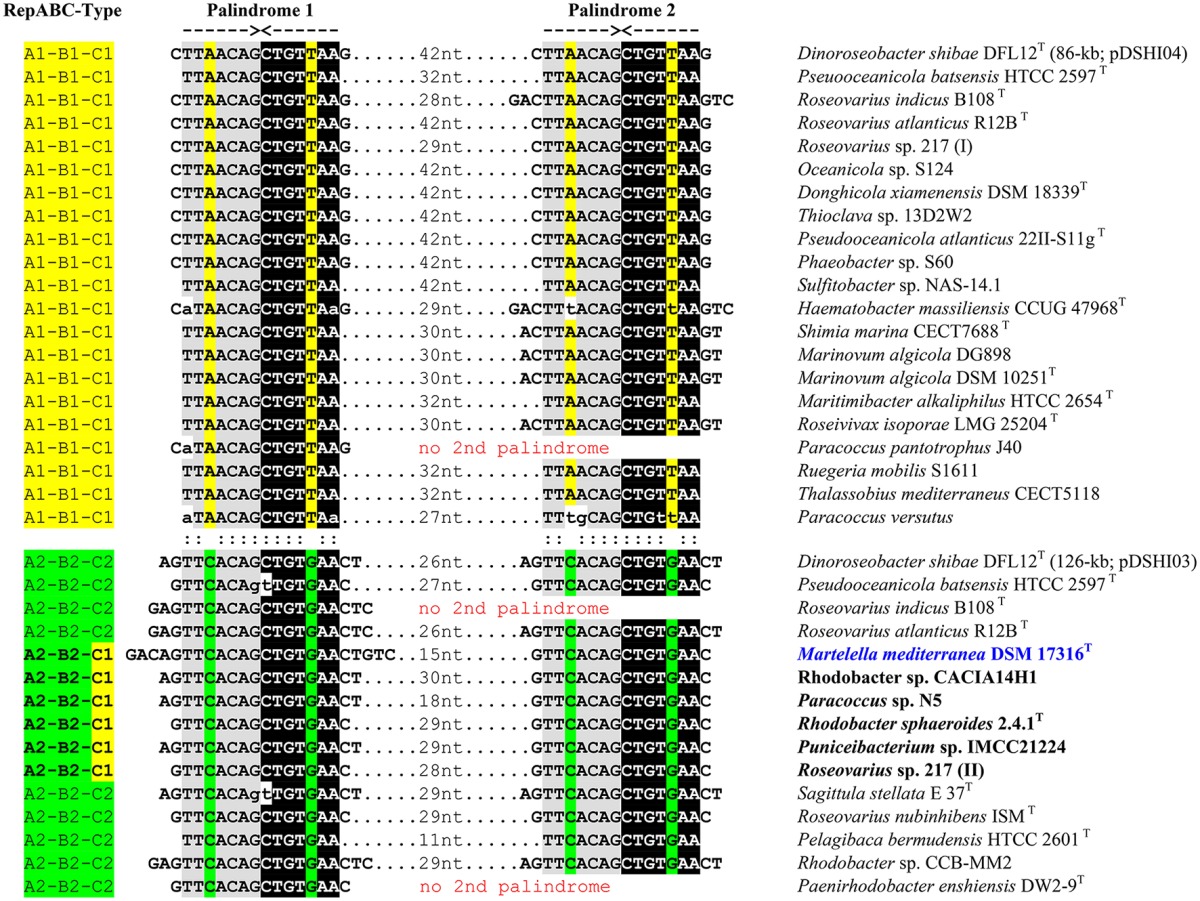
Conserved palindromes associated with RepABC-1 and -2 plasmids.

### The composite plasmid pMM259–A chimera of rhizobial and rhodobacteral ECRs

**Figure 2:**
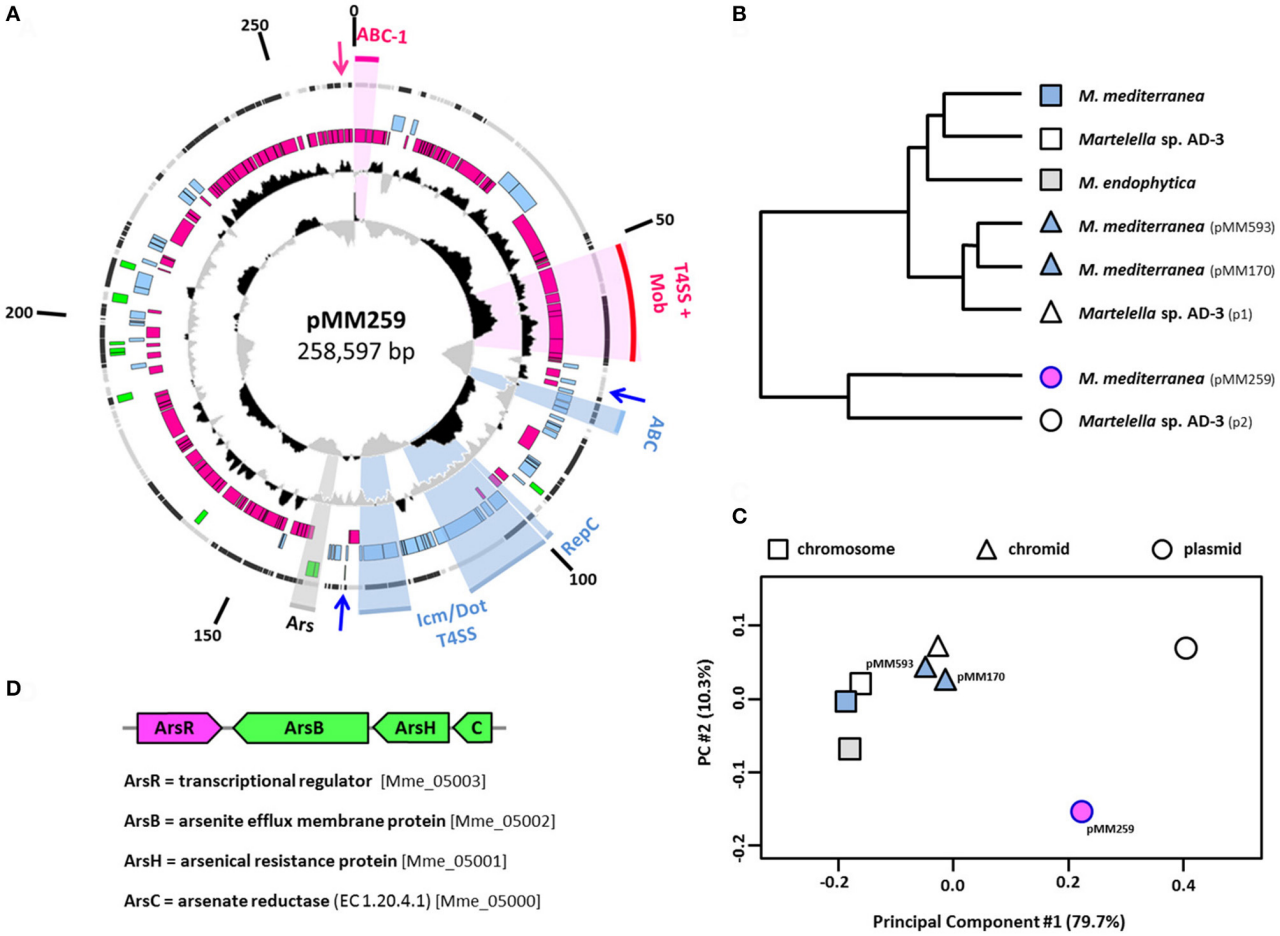
The composite *M. mediterranea* DSM 17316^T^ plasmid of mixed rhodobacteral/rhizobial ancestry. **(A)** Circular map of pMM259. Circles represent from inside to outside (1) G+C skew (10,000 bp window); (2) G+C content and deviation from the mean value (1,000 bp window) (3, 4, 5); Coding sequences (CDSs) of *Rhodobacterales*/*Rhizobiales*/other origin (pink/blue/green) (6) location on the plus or minus strand (gray/black). The origins of CDSs were determined via best BLASTP hits (*E-*value < 10^−5^). RepABC-type replication systems (ABC) and type four secretion systems (T4SS) are accentuated with sectors and labeled with respect to their origin (pink/blue). Arrows indicate the localization of toxin/antitoxin operons. Mob, mobilization module (*virD2, virD4* genes); Ars, arsenate-resistance operon. **(B,C)** Principal component and cluster analysis of relative synonymous codon usage (RSCU) based on all protein-coding sequences from the *M. endophytica* chromosome (light gray), four *M. mediterranea* (light blue), and three *Martelella* sp. AD-3 replicons (white). pMM259 is highlighted in pink. The dendrogram based on a hierarchical cluster analysis of the overall RSCU. Two-dimensional scaling explains 90.0% of the variance. Chromosomes, chromids and plasmids are indicated by squares, triangles and circles, respectively. **(D)** Structure of the arsenate-resistance operon. Xenologous genes of gammaproteobacterial origin are shown in green.

Our plasmid of interest pMM259 possesses, apart from the conspicuous rhodobacteral RepABC module (A2B2C1; see above), a complete RepABC module as well as a solitary replicase (RepC), both of rhizobial origin, thus documenting that it represents a composite plasmid with replication systems from two alphaproteobacterial orders (Figure 2A). Replicons that originate from plasmid fusion events have previously been detected in completely sequenced genomes of other rhizobia, such as *Rhizobium etli* CFN 42, *Rhizobium leguminosarum* biovar *viciae* 3841 and *Rhizobium* sp. NT-26 (González et al., 2006; Young et al., 2006; Andres et al., 2013). Both replication modules of a composite plasmid might still be functional as experimentally documented, e.g., for the 107-kb replicon of *Paracoccus versutus* UW1 (Bartosik et al., 1998). However, a second replication system is generally not required for the stable maintenance of these low copy number plasmids and can be lost again. Experimental testing of the respective modules is hence the prerequisite for determining their functionality (see below), and it allows for drawing conclusions about the intrinsic potential of plasmid fission resulting in two operative replicons.

#### Holistic classification of *Martelella mediterranea*'s extrachromosomal replicons

The “chromid” concept of Harrison et al. (2010) introduced an evolutionary dimension into the classification of ECRs based on codon usage (CU) analyses. In brief, so-called chromids are essential ECRs with a CU comparable to that of the chromosome, which mirrors their long-lasting co-evolution, whereas true plasmids are frequently exchanged via conjugation and thus exhibit a largely deviating CU (Petersen et al., 2013). We investigated the affiliation of all replicons from *M. mediterranea* DSM 17316^T^, *M. endophytica* YC6887^T^, and *Martelella* sp. AD-3, which represent the three completely sequenced genomes of this genus, in a principal component analysis (PCA) of the relative synonymous codon usage (RSCU; Figures 2B,C). The two-dimensional PCA, which explains 90.0% of the CU variance, shows a clear affiliation of pMM593 and pMM170 with *Martelella*'s chromosome, thus justifying their classification as chromids, whereas the composite replicon pMM259 represents a genuine plasmid. The capacity of horizontal exchange of this 259-kb plasmid is indicated by the presence of two T4S systems (see below) and furthermore supported by its RepABC replication system of rhodobacteral origin (A2B2C1 type; Figure 1).

#### Identification of horizontally transferred genes in pMM259

The presence of a composite plasmid with rhizobial and rhodobacteral replication systems in *M. mediterranea* indicated that this ECR might contain additional horizontally acquired genes from roseobacters. However, a reliable detection of authentic HGTs would need to be based on time-consuming phylogenetic analyses, as documented for the RepABC modules (Figure 1, Figures S2–S7). We used a customized version of HGTector (Zhu et al., 2014) as a rapid discovery tool for the detection of potential HGT-derived genes on the plasmids of *Martelella*. The program allowed us to identify many putative HGTs (Tables S1–S3), but the two chromids of *Martelella* indicate that the number of authentic vertically evolving rhizobial genes may be underestimated. HGTector proposed a comparably low number of genes that are vertically transmitted (no HGT; 41% pMM593, 49% pMM170), whereas the best BLAST hits revealed a rhizobial affiliation for a larger part of these genes (55% pMM593, 64% pMM170; Tables S1–S3). Accordingly, we used the more conservative best BLAST hits for the differentiation between vertical inherited and horizontally acquired genes (Figure 2, Figure S1).

#### Identification of rhizobial and rhodobacteral genes on the *Martelella* plasmid pMM259

Our comparison of ECRs from *M. mediterranea* recovered a genuine rhizobial affiliation for the majority of chromid-located genes [291/529 (55%) pMM593, 100/155 (65%) pMM170], but only for 29% of the genes from the composite plasmid pMM259 (69/239), whose largest portion of genes [147/239 (62%)] is of rhodobacteral origin (Figure S1). A 30-kb stretch between 183- and 213-kb on pMM259 exhibits a rather scattered distribution of rhizobial and non-rhizobial genes (Figure 2A), but many of the rhizobial genes represent transposases that might have recently been acquired by intragenomic transposition events. Accordingly, the general composition of pMM259 clearly documents that the 259-kb plasmid harbors a backbone of roseobacter-associated genes and a rhizobial insertion of about 60-kb starting upstream of the blue RepABC module and ending downstream of the Icm/Dot type T4S system [Figure 2A, Table S2 (Mame_04940 to Mame_04999)]. This spatial separation of genes with a vertical and non-vertical history indicates that the present day plasmid still reflects the fusion event of a conjugated roseobacter plasmid with a size of about 200-kb with a 60-kb equivalent from the rhizobial host. Both partners have a different nucleotide composition as illustrated by deviations of the G+C content (Figure 2A). The 60-kb remnant of the rhizobial plasmid has a remarkably low G+C content of just 58% compared to 62% in the rhodobacteral part, a proportion that is, coincidentally, comparable to those of the two chromids (62%) and the chromosome (63%). The observed difference nearly reflects the natural G+C range of rhizobial genomes, thus documenting that *Martelella* is not the natural host of the rhizobial part of pMM259. This conclusion is supported by a codon-usage subanalysis (RSCU) of the rhizobial and rhodobacteral parts of pMM259 (Figure S8). The clustering clearly documents that (i) the CUs of rhizobial and roseobacter-specific genes largely differ and that (ii) both fusion partners can be classified as plasmids with a CU largely deviating from those of the chromosome. The G+C skew plot in Figure 2A allows one to pinpoint the origin of replication of the rhizobial 60-kb fragment, which is located within the *repC* gene of the blue RepABC module (Pinto et al., 2012), and it further shows the leading and lagging strand for DNA replication (Lobry, 1996; Grigoriev, 1998). *In silico* ligation of the 60-kb fragment even allows for predicting the former terminus of replication within the *icm*/*dot* operon for pilus formation of the rhizobial T4SS. Our analyses documented that it is still possible to detect specific molecular imprints in the genuine rhizobial plasmid, thus we conclude that the plasmid fusion was, from an evolutionary point of view, a rather recent event.

#### Further xenologous genes of pMM259

The HGT analyses showed that between 4 and 11% of the genes encoded on the three ECRs from *M. mediterranea* have a distinct affiliation that is neither related to rhizobia nor to *Rhodobacteraceae* (Tables S1–S3; Figure S1). These genes are highlighted in green within the outermost colored circle of Figure 2A and Figure S1. Gene clusters with comparable best BLASTP hits are especially interesting because they indicate that whole DNA modules and not only single genes have been horizontally transferred. One example for the 259-kb plasmid is an operon with two adjacent genes from *Rhodospirallales* encoding a thiol-disulfide interchange protein precursor and a lipoprotein signal peptidase II involved in protein export (Mame_05059, Mame_05058). However, the most conspicuous finding is the xenologous arsenate-resistance operon *arsCHB* with the adjacent transcriptional regulator *arsR* (Figures 2A,D; Mame_05000-05003), which is crucial for the oxidative detoxification of the highly poisonous methylarsenite (III) to methylarsenate (V) by ArsH (Mukhopadhyay et al., 2002; Yang and Rosen, 2016). This operon is, from an evolutionary perspective, of remarkable interest, because it exemplifies that the recombination of distantly related genes from *Alpha*- and *Gammaproteobacteria* resulted in the formation of a functional unit. The transcriptional regulator ArsR originate—as indicated by its pink color—from *Rhodobacteraceae*, whereas the genes marked in green of the resistance operon have a gammaproteobacterial origin with *Halomonas zhanjiangensis* DSM 21076^T^ (*Oceanospirillales, Halomonadaceae*) as closest relative. The chronology of HGT and plasmid fusion is difficult to estimate because the module is located within the transition zone between the rhizobial and the rhodobacteral part of plasmid pMM259 (Figure 2A). The horizontal transfer of the gammaproteobacterial *arsCHB* operon might thus either reflect a rather recent event in the genus *Martelella* or it already occurred within roseobacters prior to plasmid conjugation.

### Origin and distribution of the composite plasmid

#### Type IV secretion systems of the composite plasmid pMM259

We investigated the origin of the two type IV secretion systems (T4SS) located on the composite 259-kb plasmid (Figure 2A) based on the assumption that one of them might have mediated the conjugational transfer of the roseobacter-specific A2B2C1-type RepABC plasmid into *M. mediterranea* (Figure 1, Table 1). The superoperon marked in blue of rhizobial origin represents an Icm/Dot T4SS with characteristic *icm* and *dot* genes (Mame_04961-04992; Juhas et al., 2008). This extremely divergent secretion system harbors a conjugative transfer relaxase gene *traA* (Mame_04988; Table S2), thus indicating that it is responsible for plasmid conjugation, whereas comparable conserved systems of *Legionella pneumophila* and *Coxiella burnetii* are utilized for bacterial pathogenesis (Segal et al., 2005). Syntenous superoperons including the mobilization genes have been identified in other rhizobia, such as *Sinorhizobium* sp. CCBAU 05631 or *Ochrobactrum anthropi* OAB. We were surprised that the “pink” T4S system of rhodobacteral origin also contains all essential genes for conjugational plasmid transfer. Its module structure, which comprises the *virB* secretion apparatus (Mame_04915-04935) and a cluster of mobilization genes including the crucial relaxase and the coupling protein [*virD2* (Mame_04917), *virD4* (Mame_04918)], is absolutely conserved regarding homologs from other *Rhodobacteraceae* (Petersen et al., 2013). One example is the duplicated T4SS from the 191-kb and 126-kb sister plasmids of *D. shibae* DFL12^T^, whose conjugation across genus barriers has recently been demonstrated (Patzelt et al., 2016).

Our analyses suggest that the “pink” T4S system mediated plasmid conjugation from a still unknown roseobacter (donor) into the rhizobial recipient *Martelella*, thus explaining the large portion the rhodobacteracean genes on pMM259 (Figure 2A). Moreover, the structural integrity of the investigated T4SS systems indicates that the 259-kb plasmid from *Martelella* is still conjugative. Accordingly, horizontally transferred syntenous plasmids are waiting to be discovered in other marine bacteria. This aim seems to be like searching for a needle in the haystack, but horizontal plasmid transfer in the ocean has—concomitant with the exponential increase of whole genome sequences—very recently been reported for two roseobacters i.e., *D. shibae* DFL12^T^ and *Confluentimicrobium naphthalenivorans* NS6^T^ (Petersen and Wagner-Döbler, 2017).

#### The closest relative of the composite plasmid pMM259

**Figure 3:**
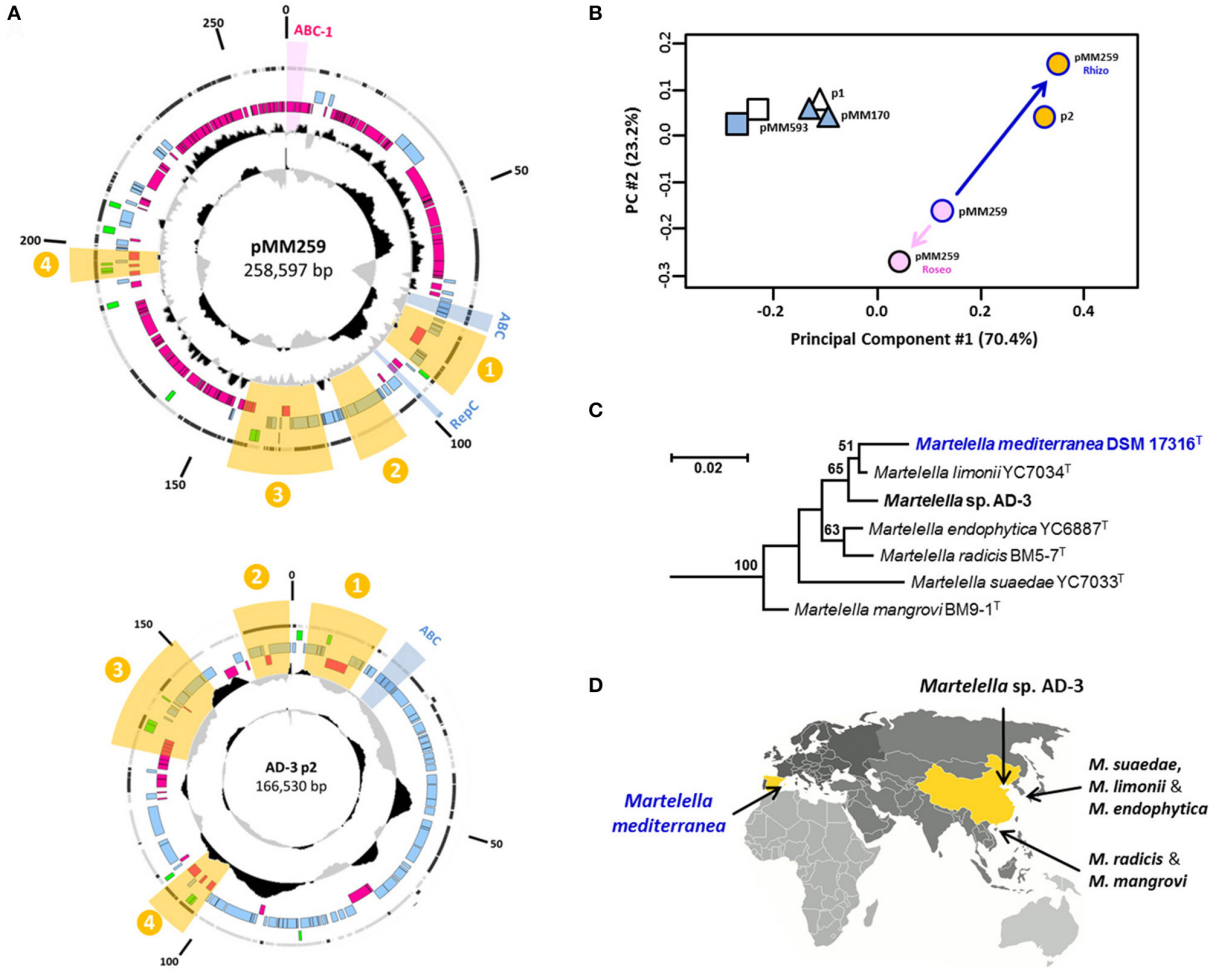
Comparison of plasmids from *M. mediterranea* DSM 17316^T^ and *Martelella* sp. AD-3. **(A)** Circular maps of pMM259 and p2. Syntenous regions are highlighted with yellow sectors. **(B)** RSCU analysis; rhizobial and rhodobacteral parts of pMM259 were investigated separately. **(C)** 16S-rRNA gene analysis of all six type strains and the isolate AD-3 of the genus *Martelella*. **(D)** Provenance of *Martelella* strains based on the place of isolation.

The conspicuous separation of rhodobacteral and rhizobial genes on the composite plasmid is indicative of a rather recent fusion event (see above; Figure 2). Accordingly, we tried to identify close syntenous relatives of pMM259 with BLASTN searches in the non-redundant (nr) and whole-genome shotgun (wgs) nucleotide databases of the NCBI. This approach allows for the identification of conserved genetic modules and is based on the detection of silent mutations more sensitive than a standard BLASTP search. However, our analyses revealed no highly specific *Rhodobacteraceae* hits with more than 95% sequence identity. This outcome documents that the donor for plasmid fusion is still undetected, which is in agreement with the phylogenetic analyses of the A2B2C1 plasmid-replication module (see above). In contrast, the composite *M. mediterranea* plasmid pMM259 specifically matches with the 167-kb plasmid “p2” from *Martelella* sp. AD-3 (CP014277.1), and the four syntenic regions with a total size of 47-kb exhibit an average sequence identity between 95 and 99% (highlighted in yellow, Figure 3A). Their close affiliation is independently shown by the RSCU comparison of plasmid p2 with the rhizobial part from pMM259 (Figures 3B,C). Three of the conserved areas including region “three,” which contains the arsenate-resistance operon (Figure 2D), are matching the rhizobial part of *M. mediterranea*'s 259-kb plasmid, but region “four” shares 97% sequence identity with the rhodobacteral part of pMM259. This distribution indicates that the 167-kb plasmid from *Martelella* sp. AD-3 might also originate from the composite rhizobial/rhodobacteral fusion plasmid and secondarily lost the majority of roseobacter-specfic genes including the A2B2C1-type RepABC replication module and the T4SS.

### pMM259–A natural plasmid for horizontal gene transfer between *Rhodobacteraceae* and *Rhizobiaceae*

#### Rationale for the experiments

The presence of two complete RepABC-type replication systems of rhizobial and rhodobacteral origin on the composite *M. mediterranea* plasmid is of particular interest because it suggests that pMM259 might represent a natural replicon mediating HGT between two alphaproteobacterial orders. Accordingly, we established a transformation assay that allowed us to monitor the replication of RepABC-type plasmid modules based on antibiotic selection and chose the model organisms *P. inhibens* DSM 17395 and *A. tumefaciens* C58 DSM 5172 (synonyms *Agrobacterium radiobacter, Rhizobium radiobacter*; Tindall, 2014) as test strains.

#### Cloning of RepABC modules

**Figure 4:**
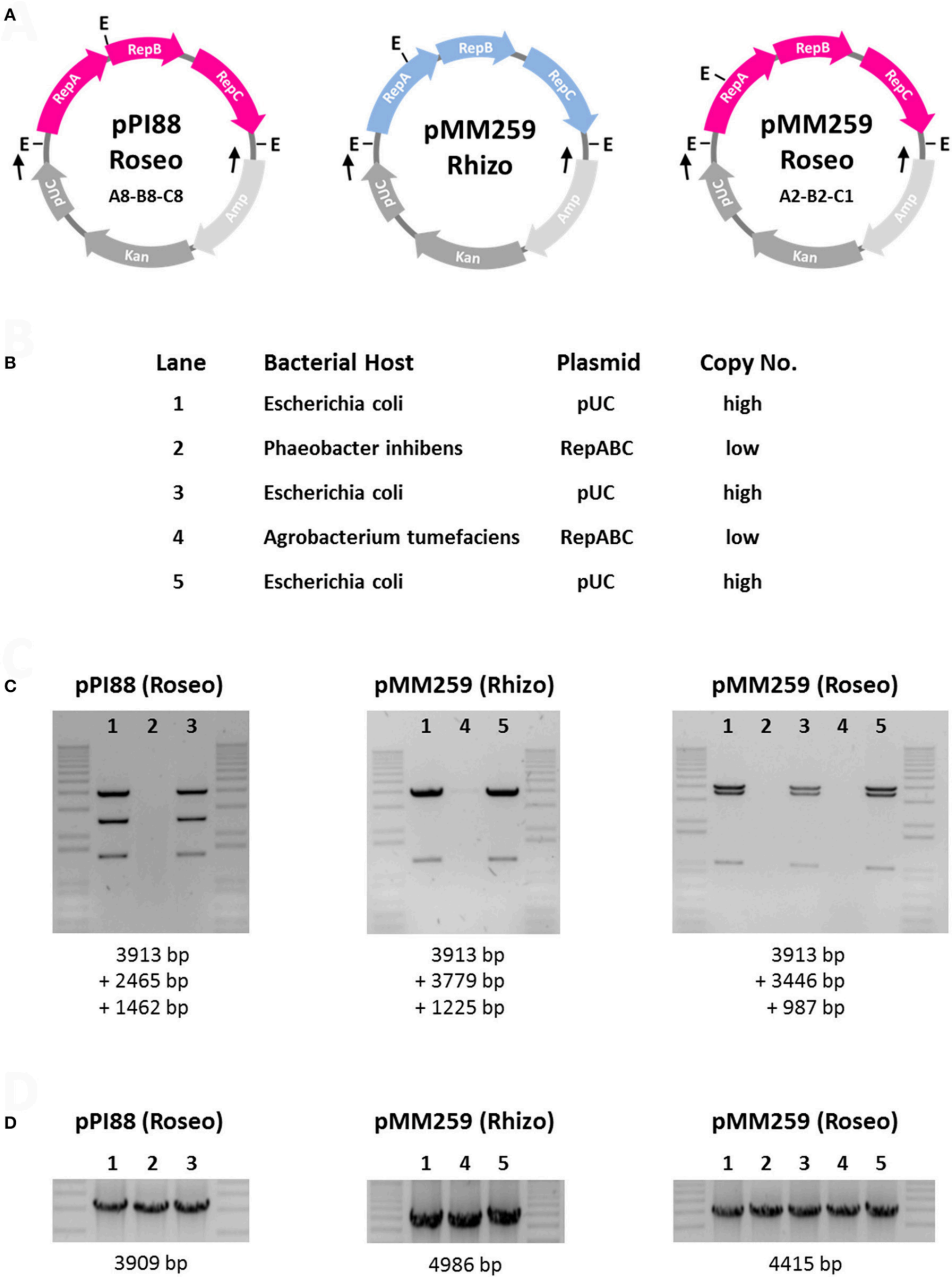
Functionality tests of rhizobial and rhodobacteral RepABC-type plasmids in *Phaeobacter inhibens* DSM 17395 and *Agrobacterium tumefaciens* C58. **(A)** Schematic plasmid maps of the tested constructs. Roseobacter and rhizobial RepABC-type replication systems are shown in pink and blue, respectively. The genes of the cloning vector pCR2.1 are shown in gray. pUC, origin of replication; Kan and Amp, kanamycin and ampicillin resistance genes; E, *Eco*RI restriction site; Black arrows indicate primer binding sites for PCR amplification. **(B)** Specificity and copy number of composite plasmids based on the host range of the plasmid replication system. **(C)** Plasmid restriction assay with *Eco*RI. The 1 kb Plus DNA ladder from Invitrogen was used as a marker. **(D)** PCR assay with pCR2.1-specific primers.

Two RepABC operons from the 259-kb *M. mediterranea* plasmid, i.e., the roseobacter-specific RepABC module (4415 bp; A2B2C1) and the genuine rhizobial RepABC module (4986 bp), were cloned into the commercial vector pCR2.1 (see Experimental Procedures). Furthermore, we searched for a rhodobacteral positive control for our stability tests and analogously cloned the RepABC-8 operon from *P. inhibens* T5^T^ (DSM 16374^T^; 3909 bp, A8B8C8). The respective module is specific for the type strain and located on an 88-kb plasmid, which is missing in other isolates, such as *P. inhibens* DSM 17395 (Thole et al., 2012; Dogs et al., 2014). The resulting plasmids pPI88-Roseo, pMM259-Rhizo and pMM259-Roseo that are shown in Figure 4A represent artificial shuttle vectors with a host-specific copy number. In *Escherichia coli* (*Enterobacteriaceae, Gammaproteobacteria*) they replicate based on the modified pUC origin derived from a ColE1/pMB1 vector as high copy number plasmids [500–700 copies per chromosome (HCNP); Gelfand et al., 1978; Lee et al., 2006], in contrast to the alphaproteobacterial host(s) where the respective RepABC system ensures a stable maintenance as a low copy number plasmid [1 copy per chromosome (LCNP); Pappas, 2008].

#### Functionality tests of RepABC modules in *P. inhibens* DSM 17395 and *A. tumefaciens* C58

*Phaeobacter inhibens* DSM 17395 and *A. tumefaciens* C58 are both sensitive to the antibiotic kanamycin, and we accordingly used the respective resistance gene of pCR2.1 as a selection marker for our experiments (Figure 4A). Transformation of a circular pCR2.1 plasmid without an insert was used as negative control and confirmed that the *E. coli* cloning vector does not replicate in *Alphaproteobacteria*. A potential pitfall of the functionality test is a stable integration of the pCR2.1 construct into the chromosome, which would also result in kanamycin-resistant transformants. Accordingly, and as an ultimate proof of functional plasmid replication, we isolated the LCNPs from the alphaprotebacterial host, retransformed them into *E. coli* and showed that the *Eco*RI restriction patterns of isolated plasmid DNA are identical to those of the original digests (lane 1&3, lane 1&5; Figure 4C). The absence of DNA fragments in lanes 2 and 4 mirrors the low copy number of RepABC-type plasmids in *Alphaproteobacteria* but control PCRs showed that the respective LCNPs are present in all three (five) samples (Figure 4D).

Based on this experimental setup, we were able to document the functionality of our assay including the selected test strains. The positive control pPI88-Roseo mediated—based on its RepABC-8 module—stable plasmid replication in *P. inhibens* DSM 17395, but it does not replicate in *A. tumefaciens* C58 (Figure 4). The rhizobial RepABC module from *Martelella* (pMM259-Rhizo) showed a reciprocal pattern and is only replicated in *Agrobacterium*. This finding is in agreement with the strict phylogenetic separation of rhizobial and rhodobacteral RepABC replication systems (Figure 1), which led to the *in silico* prediction of functional incompatibility (Petersen et al., 2009). Furthermore, our assay did not only validate the functionality of *Martelella*'s xenologous A2B2C1 plasmid replication system in *Phaeobacter*, it surprisingly also showed that pMM259-Roseo is—at least under kanamycin selection—replicated and stably maintained in *A. tumefaciens* (Figures 4C,D). The outcome is contradictory to the experiments with the RepABC-8 operon of *P. inhibens* T5^T^ and indicates that some RepABC-type plasmids of *Rhodobacteraceae* might have a broader host range than previously assumed. This prediction is supported by the presence of a rhodobacteral operon on the composite 322-kb plasmid from *Rhizobium* sp. NT-26 (Andres et al., 2013) but especially by former host-range tests with the RepABC-1 operon of the composite 107-kb plasmid pTAV1 from *P. versutus* UW1, which documented stable replication in *Rhizobium etli* CE3 and *Rhizobium leguminosarum* 1062 (Bartosik et al., 1998). In contrast, the rhizobial RepABC module from *Martelella* (pMM259-Rhizo; Figure 4) showed the expected host range limited to rhizobia. This outcome was independently validated by the respective operon from the Ti-plasmid (*A. tumefaciens* C58) that does also not replicate in *P. inhibens* DSM 17395 (data not shown), thus indicating that functional constraints prevent the replication of rhizobial RepABC plasmids in *Rhodobacteraceae*.

#### Stability tests of RepABC modules replicating in *Phaeobacter* and *Agrobacterium*

The presence of two replication systems on *Martelella*'s 259-kb plasmid is surprising, because the rhizobial module should be sufficient for replication. Accordingly, we proposed that the stability of pMM259-Roseo is reduced in rhizobia and tested this hypothesis experimentally based on the four previously established transformants (Figure 4). The tests of pPI88-Roseo and pMM259-Roseo in *Phaeobacter*, which served as a reference, showed that about 5% of the cells, i.e., two of 40 tested colonies, lost their RepABC-type plasmid over night during exponential growth under non-selective growth conditions (Figure S9). The comparable stability of both constructs documented that the roseobacter-specific module from *Martelella* is not only functional in *Rhodobacteraceae*, it is even unaffected in its viability. The outcome of analogous tests with the two *Martelella* constructs pMM259-Roseo and pMM259-Rhizo in *Agrobacterium* was completely unexpected, because it showed that 90% of the host cells lost the genuine rhizobial RepABC construct spontaneously (36/40 colonies), whereas the xenologous rhodobacteral module was lost in just one of the 40 tested colonies (2.5%; Figure S9). We validated the presence of the respective construct for two resistant colonies to exclude any sample mix up and repeated the experiment, which resulted in comparable rates of spontaneous plasmid loss (pMM259-Roseo: 0/40; pMM259-Rhizo: 32/40). Yet, pMM259-Rhizo is still functional and maintained in *Agrobacterium* under selective pressure, but the high frequency of loss under non-selective growth conditions might reflect an ongoing degeneration of the RepABC-system into a “pseudogene module.” Accordingly, the most probable evolutionary scenario predicts that the selective pressure exclusively remains on the functional rhodobacteral RepABC cassette. The inactivated rhizobial module will get lost soon thus erasing the plasmid-specific molecular footprint of one fusion partner in the composite plasmid pMM259.

The rate of plasmid loss observed in the current study correlates with an exponential growth of the host cell in extremely nutrient-rich medium and is thus not representative for the natural habitat. Stable maintenance of natural plasmids is promoted by beneficial and sometimes even essential genes and furthermore ensured by toxin/antitoxin systems (Zielenkiewicz and Cegłowski, 2001), which is exemplified by three respective modules on pMM259 (Figure 2). Taken together, the replication module pMM259-Roseo has a broader host range than its equivalent pMM259-Rhizo (Figure 4) and it moreover showed an unexpected stability in both tested host strains [*Phaeobacter* (*Rhodobacteraceae*), *Agrobacterium* (*Rhizobiaceae*); Figure S9]. Accordingly, this A2B2C1-cassette represents the “functional heart” of a natural plasmid that should mediate stable genetic exchange between alphaproteobacterial orders essentially based on the presence of two different T4SSs (Figure 2).

#### Significance of the composite plasmid pMM259 and conclusion

In the current study we established the complete genome sequence of the rhizobium *M. mediterranea* DSM 17316^T^. Its composite 259-kb replicon, which originated from a plasmid fusion, still harbors two functional RepABC modules that ensure plasmid replication in *Rhizobiaceae* and *Rhodobacteraceae* (Figures 1, 2, 4). *M. mediterranea* has been isolated from the subterreanean Lake Martel in the Dragon Cave on the Spanish island Mallorca (Rivas et al., 2005). This saline karst lake is located in very close proximity of the Mediterranean Sea and represents an ideal location for the intimate contact of a halotolerant rhizobium with roseobacters. Accordingly, there are no ecological boundaries preventing trans-order conjugation. The presence of two conserved T4S systems strongly indicates that pMM259 is still mobilizable (Figure 2), and it is thus likely that it mediates HGT from the globally occurring marine genus *Martelella* (Figure 3) into new rhizobial as well as roseobacter recipients. This plasmid is to our knowledge the first example of a natural replicon bridging the phylogenetic gap between these alphaproteobacterial orders. The presence of two replication systems on pMM259 overcomes the problem of the narrow-host-range of the rhizobial RepABC-type plasmids, and we thus propose that analogous plasmid fusions facilitate the genetic exchange even between bacterial classes. Previously, an outsourcing of the complete photosynthesis gene cluster for aerobic anoxygenic photosynthesis (AAnP) from the chromosome to a plasmid has been documented within the genus *Roseobacter* (Petersen et al., 2012). According to the “Think Pink” scenario (Petersen et al., 2013), plasmid conjugation could explain the presence of a homologous superoperon for AAnP in the marine gammaproteobacterium *Congregibacter litoralis* KT71^T^ (Fuchs et al., 2007). Natural shuttle vectors would hence connect distantly related bacterial lineages from the same habitat thereby providing access to the metabolic potential of the marine pan-genome.

## Experimental procedures

### Bacterial strains, plasmids, and growth conditions

Bacterial strains and plasmids used in this study are listed in Table S4. For preparation of competent cells and isolation of genomic DNA all *Rhodobacteraceae* and *Rhizobiaceae* strains were cultured in 40 g/l Marine Broth medium (MB, Carl Roth) at 28°C and 120 rpm. ½ MB with 120 μg/ml kanamycin (Carl Roth) was used for antibiotic selection.

### Host range tests of RepABC replication systems

The RepABC replication systems of *P. inhibens* T5^T^ (= DSM 16374^T^) and *M. mediterranea* DSM 17316^T^ were amplified from genomic DNA by PCR using the specific primers P1093 (5′-ACCGGCGACACAACACTCACC-3′) and P1094 (3′-ACGCGTGATCTTTCTGCTCTT-5′) for pPI88-Roseo, P1245 (5′-CGTCGAGCAGGTAAAGAACG-3′) and P1246 (3′-GTTTCGACCCCTTCAGCATC-5′) for pMM259-Roseo and P1289 (5′-GCTCATCGTACCGTTTGTCC-3′) and P1290 (3′GCGAAATCCACGGTAATGCT-5′) for pMM259-Rhizo with the Phusion proof-reading polymerase (Thermo-Fischer Scientific). The obtained PCR products were subsequently cloned into the *E. coli* vector pCR2.1 with a kanamycin resistance and a pUC origin of replication, which is not functional in *Alphaproteobacteria*. Control sequencing documented the integrity of the modules and the absence of PCR errors. We chose *P. inhibens* DSM 17395 (*Rhodobacteraceae*) and *A. tumefaciens* C58 DSM 5172 (*Rhizobiaceae*) as representative hosts for plasmid stability experiments. Electrocompetent cells were generated as previously described (Dower et al., 1988). Electroporation was conducted using 50 ng plasmid DNA in a 2 mm cuvette and 2.5 kV. Colonies grown were passaged three times on fresh agar plates under constant antibiotic pressure to eradicate residual untransformed plasmids from the culture. Plasmid DNA was isolated with the NucleoSpin Plasmid kit from Macherey-Nagel. PCR with the generic pCR2.1 vector primers P022 (5′-GGAAACAGCTATGACCATGATTAC-3′) and P023 (5′-CGTAATACGACTCACTATAGGGC-3′) was performed to detect low copy number plasmids. Retransformation of the isolated plasmid DNA into *E. coli* allowed for excluding false positives resulting from genomic integration of the kanamycin resistance gene and thus to verify the functionality of the tested RepABC replication systems. The integrity of retransformed constructs was documented by *Eco*RI digestion and gel electrophoresis.

### Stability tests of RepABC replication systems

Bacterial transformants (*P. inhibens, A. tumefaciens*) harboring RepABC modules cloned in pCR2.1 were grown in a test tube with 3 ml MB medium and kanamycin (120 μg/ml) overnight. 10 μl of the culture was transferred in a 50 ml Erlenmeyer flask with 10 ml MB medium without antibiotics and grown for 16 h. The cultures were streaked out on MB plates and incubated for 2 days. Single colonies have been resuspended in 20 μl MB medium and 3 μl of these cells were in parallel spotted on MB plates with and without kanamycin. We investigated the presence of 40 independent colonies of each transformant and could thus monitor the stability of the RepABC-type plasmid in the respective host bacterium.

### PacBio library preparation and sequencing

A SMRTbell™ template library was prepared according to the instructions from PacificBiosciences, Menlo Park, CA, USA, following the Procedure & Checklist- >10 kb Template Preparation Using Ampure® PB Beads. Briefly, for preparation of 10 kb libraries 8 μg genomic DNA was sheared using g-tubes™ from Covaris, Woburn, MA, USA according to the manufacturer's instructions. DNA was end-repaired and ligated overnight to hairpin adapters applying components from the DNA/Polymerase Binding Kit P6 from Pacific Biosciences, Menlo Park, CA, USA. Reactions were carried out according to the manufacturer's instructions. BluePippin™ Size-Selection to 7 kb was performed according to the manufacturer's instructions (Sage Science, Beverly, MA, USA). Conditions for annealing of sequencing primers and binding of polymerase to purified SMRTbell™template were assessed with the Calculator in RS Remote, PacificBiosciences, Menlo Park, CA, USA. SMRT sequencing of two SMRT cells was carried out on the PacBio *RSII* (PacificBiosciences, Menlo Park, CA, USA) taking 240-min movies.

### Genome assembly, error correction, and annotation

*De novo* genome assembly of *M. mediterranea* DSM 17316^T^ was carried out based on 67,093 post-filtered PacBio reads with an average read length of 13,478 bp using the “RS_HGAP_Assembly.3” protocol included in SMRT Portal version 2.3.0 applying default parameters. The assembly process revealed one circular chromosome and three ECRs. End trimming and circularization was performed, where the chromosome was adjusted to *dnaA* and all ECRs to their replication genes. Finally, each genome was error-corrected by a mapping of Illumina reads onto finished genomes using BWA (Li and Durbin, 2009) with subsequent variant and consensus calling using VarScan (Koboldt et al., 2012). Correct replicon structures and a consensus concordance of QV60 were confirmed by using the “RS_Bridgemapper.1” protocol. Finally, an annotation was generated using Prokka 1.8 with subsequent manual reannotation of all replication genes (Seemann, 2014). Complete genomes were deposited at NCBI GenBank under the accession numbers CP020330 to CP020333.

### Analysis of horizontally transferred genes

HGT analysis of *M. mediterranea* DSM 17316^T^ plasmids was conducted using HGTector.py (Zhu et al., 2014) with BLASTP against the NCBI non-redundant (nr) sequence database (download: October, 12th 2016), the taxonDMP (October, 12th 2016) and release 78 of MultispeciesAutonomousProtein2taxname from RefSeq. To exclude self-hits the corresponding TaxID (293088) was defined as self-group of *M. mediterranea*. The close group was defined as TaxID 356 (*Rhizobiales*) respectively, all other organisms in the nr database made up the distal group. Best hits from up to 500 blast results with a 10^−5^ *e*-value cutoff were used to determine the origin of genes on order level. Further analysis and creation of circle plots was accomplished by custom R scripts utilizing ggbio, GRanges, ggplot2, rentrez, and taxize packages.

### Phylogenetic analyses

The amino acid and nucleotide alignments of RepABC genes obtained with ClustalW (Thompson et al., 1997) were manually refined using the ED option of the MUST program package (Philippe, 1993). Gblocks was used to eliminate both highly variable and/or ambiguous portions of the alignments (Talavera and Castresana, 2007). Maximum likelihood (ML) analyses were performed with RAxML version 8.2.4 (Stamatakis, 2014) applying Pthreads to use multiple shared memory nodes and SSE3 vector instructions, which together allow for substantially speeding up the computations depending on the number of nodes used. In RAxML a rapid bootstrap analysis with 100 replicates followed by a thorough search of the ML tree was conducted under the LG+F+4Γ model. For protein analyses of the RepABC modules the neighbor-joining algorithm with gamma-corrected distances under the JTT model including 100 bootstrap replicates was used as described in Petersen et al. (2011). The calculations were performed in the program MEGA version 5 (Tamura et al., 2011) in an interactive way via the graphical user interface (GUI).

## Author contributions

JP and PB designed research. PB, HB, and BB contributed new data. PB, HB, BB, and JP performed analyses. BB and MG contributed software tools. JP, HB, and PB drafted manuscript and all authors read and approved the final manuscript.

### Conflict of interest statement

The authors declare that the research was conducted in the absence of any commercial or financial relationships that could be construed as a potential conflict of interest.

## Supplementary material

Figure S1 Extrachromosomal replicons of *M*. *mediterranea* DSM 17316^T^. Circles represent from inside to outside (1) G+C Skew (10,000 bp window); (2) G+C content and deviation from the mean value (1,000 bp window); (3, 4, 5) Coding sequences (CDSs) of *Rhodobacterales*/*Rhizobiales*/other origin (pink/blue/green); (6) location on plus or minus strand (gray/black). The origins of CDSs were determined via best BLASTP hits (*E-*value < 10^−5^). The actual scale between plasmids is not taken into account. Toxin/antitoxin operons for plasmid stability are indicated by stars.

Figure S2 Composite Neighbor Joining tree of 121 **RepC** replicases from RepABC-type plasmids representing all nine *Rhodobacteraceae*-specific **compatibility groups** (C1 to C9). The upper subtree based on 50 sequences from *Rhodobacteraceae* and 145 amino acid position (α = 0.92; JTT) and the lower subtree based on 71 mostly rhizobial sequences including rhodobacteracean RepC-9 proteins and 226 amino acid positions (α = 1.03; JTT). The statistical support for the internal nodes was determined by 100 bootstrap replicates (BR) and values >50% are shown. Internal rooting was performed according to the RepC-tree of Petersen et al. (2009). Rhodobacteracean subtrees and the “rhizobial” tree are highlighted by pink and blue boxes, respectively.

Figure S3 Neighbor Joining tree (p-distances; 100 BR) of **RepA** partitioning proteins from the RepABC plasmid replication operon of the rhodobacteracean compatibility groups 1 and 2 based on 81 sequences and 386 amino acid positions. *Martelella mediterranea* DSM 17316^T^ is highlighted in blue. Strains for phylogenetic subanalyses are highlighted in green (Figure 1B, Figure S5).

Figure S4 Neighbor Joining tree (p-distances; 100 BR) of **RepB** partitioning proteins from the RepABC plasmid replication operon of the rhodobacteracean compatibility groups **1** and **2** based on 81 sequences and 225 amino acid positions.

Figure S5 Neighbor Joining tree (p-distances; 100 BR) of **RepC** replicases from the RepABC plasmid replication operon of the rhodobacteracean compatibility groups **1** and **2** based on 81 sequences and 352 amino acid positions.

Figure S6 Phylogenetic positioning of the *repAB* partitioning module from *Martelella mediterranea* DSM 17316^T^. Strains with A2B2C1-type plasmid replication systems (Figures S2–S4) are highlighted in bold and green. **(A)** Maximum Likelihood [ML] tree (RAxML, LG+F+4Γ; 100 BR) of **RepA2** proteins based on nine sequences and 394 amino acid positions. **(B)** ML tree (RAxML, LG+F+4Γ; 100 BR) of **RepB**2 proteins based on 312 amino acid positions. **(C)** ML tree (RAxML, LG+F+4Γ; 100 BR) of concatenated **RepA2** and **RepB2** proteins based on 706 amino acid positions. **(D)** ML tree (RAxML, GTR+4Γ; 100 BR) of concatenated ***repA2*** and ***repB2*** genes based on 2164 nucleotide positions.

Figure S7 Phylogenetic positioning of the *repC* replication gene from *Martelella mediterranea* DSM 17316^T^. Strains with A2B2C1-type plasmid replication systems (Figures S2–S4) are highlighted in bold and green. **(A)** Maximum Likelihood tree (ML; RAxML, LG+F+4Γ; 100) of **RepC1** proteins based on 39 sequences and 403 amino acid positions. **(B)** ML tree (RAxML, LG+F+4Γ; 100 BR) of RepC1 proteins based on 17 sequences and 402 amino acid positions. **(C)** ML tree (RAxML, GTR+4Γ; 100 BR) of ***repC1*** genes based on 17 sequences and 1212 nucleotide positions.

Figure S8 Principal component and cluster analysis of relative synonymous codon usage (RSCU) based on all protein-coding sequences from the four *M. mediterranea* replicons. The rhodobacteral (Roseo) and rhizobial (Rhizo) specific genes of pMM259 were also analyzed separately. Their distribution is highlighted in the plasmid map in pink or in blue, respectively. Two-dimensional scaling explains 98.0% of the variance. Chromosomes, chromids and plasmids are indicated by squares, triangles and circles, respectively.

Figure S9 Plasmid stability tests of the rhizobial and rhodobacteral RepABC-type plasmids pMM259-Roseo, pMM259-Rhizo and pPI88-Roseo in *Phaeobacter inhibens* DSM 17395 and *Agrobacterium tumefaciens* C58 (see Figure 4). 3 μl of resuspended bacterial colonies were in parallel spotted on two agar plates with and without the antibiotic kanamycin.

Table S1 HGT Analysis of *Martelella mediterranea* plasmid pMM593.

Table S2 HGT Analysis of *Martelella mediterranea* plasmid pMM259.

Table S3 HGT Analysis of *Martelella mediterranea* plasmid pMM170.

Table S4 Strains and vectors used in this study.
