# Supplementary material for: The Composite 259-kb Plasmid of Martelella mediterranea DSM 17316T–A Natural Replicon with Functional RepABC Modules from Rhodobacteraceae and Rhizobiaceae
Source: Front Microbiol. 2017 Sep 21;8:1787. doi: 10.3389/fmicb.2017.01787 (PMC5613091; doi:10.3389/fmicb.2017.01787)
Supplement: Figure S1 — Extrachromosomal replicons of M. mediterranea DSM 17316T. Circles represent from inside to outside (1) G+C Skew (10,000 bp window); (2) G+C content and deviation from the mean value (1,000 bp window); (3, 4, 5) Coding sequences (CDSs) of Rhodobacterales/Rhizobiales/other origin (pink/blue/green); (6) location on plus or minus strand (gray/black). The origins of CDSs were determined via best BLASTP hits (E-value < 10−5). The actual scale between plasmids is not taken into account. Toxin/antitoxin operons for plasmid stability are indicated by stars. [file Image1.PDF]

Fig. S1

ECRs of *Martelella mediterranea* DSM 17316^T

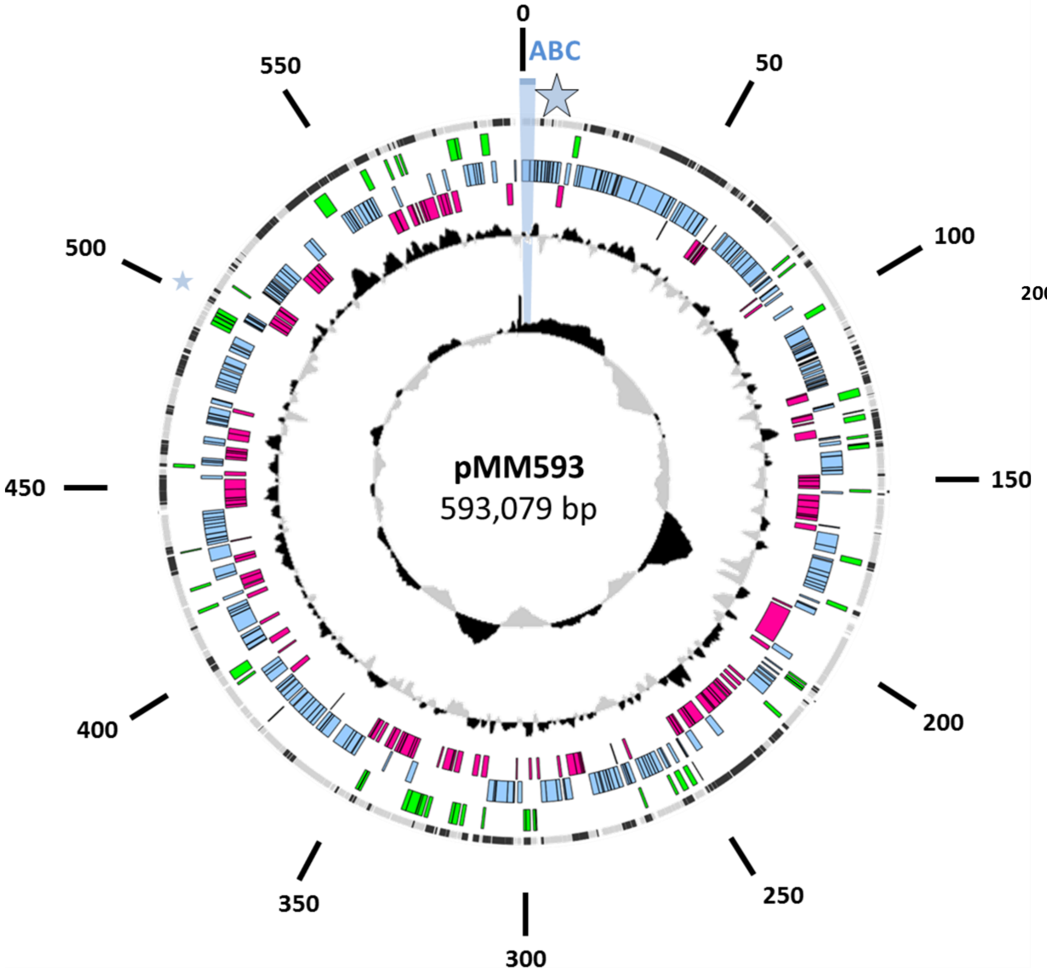

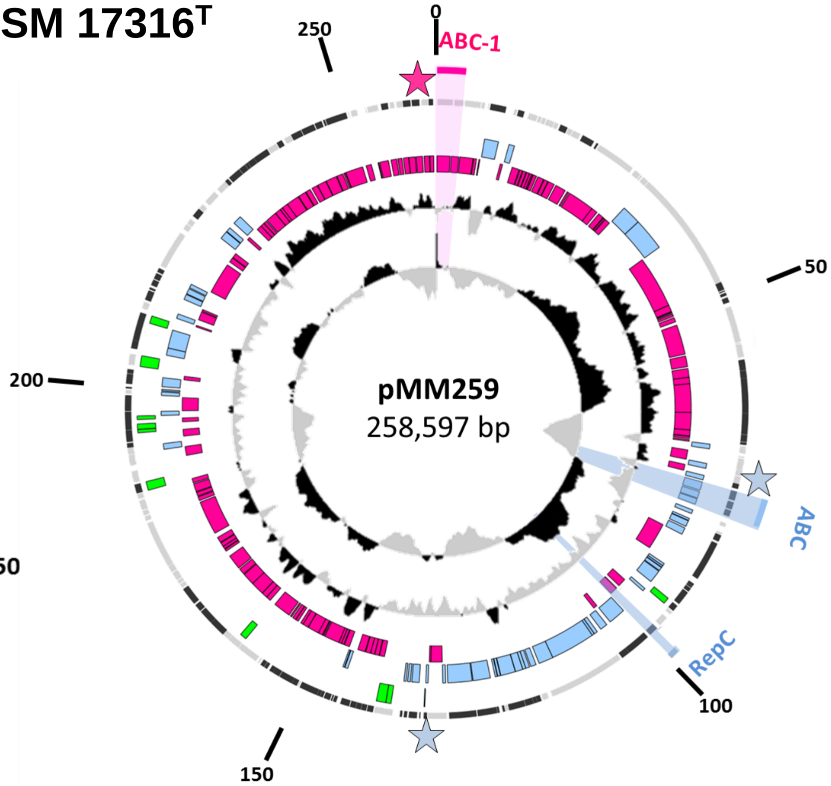

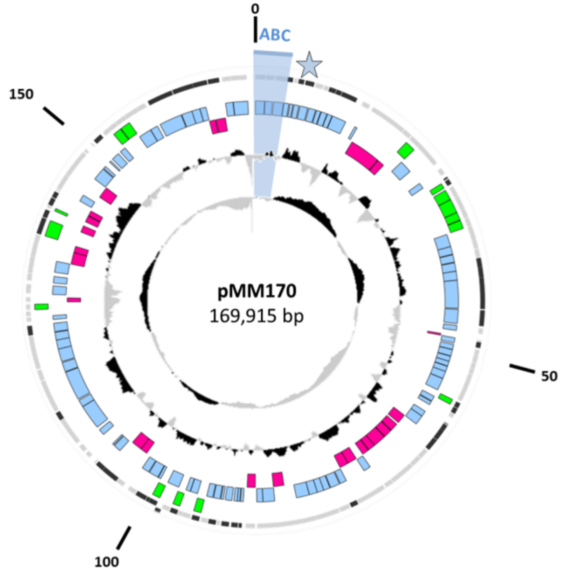

	pMM593	pMM259	pMM170
Protein coding genes	529	239	155
Rhizobiales origin	291 (55%)	69 (29%)	100 (65%)
Rhodobacterales origin	142 (27%)	147 (62%)	28 (18%)
Other origin	59 (11%)	10 (4%)	17 (11%)
NA	37 (7%)	13 (5%)	10 (6%)
