# Supplementary figures and images for: The Composite 259-kb Plasmid of Martelella mediterranea DSM 17316T–A Natural Replicon with Functional RepABC Modules from Rhodobacteraceae and Rhizobiaceae
Source: Front Microbiol. 2017 Sep 21;8:1787. doi: 10.3389/fmicb.2017.01787 (PMC5613091; doi:10.3389/fmicb.2017.01787)

Fig. S2 RepC-Phylogeny (C1-C9)

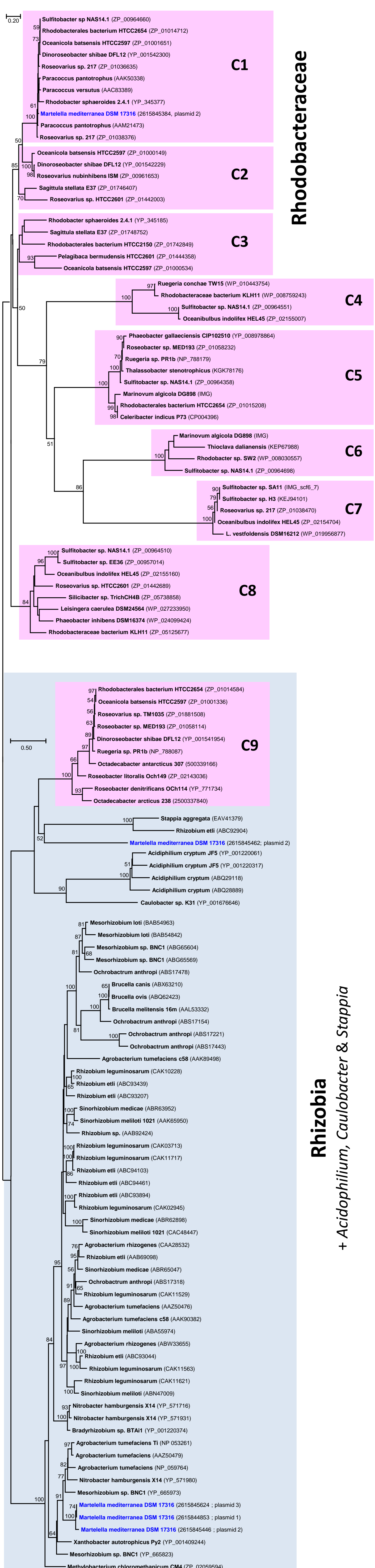

Supplement: Figure S2 — Composite Neighbor Joining tree of 121 RepC replicases from RepABC-type plasmids representing all nine Rhodobacteraceae-specific compatibility groups (C1 to C9). The upper subtree based on 50 sequences from Rhodobacteraceae and 145 amino acid position (α = 0.92; JTT) and the lower subtree based on 71 mostly rhizobial sequences including rhodobacteracean RepC-9 proteins and 226 amino acid positions (α = 1.03; JTT). The statistical support for the internal nodes was determined by 100 bootstrap replicates (BR) and values >50% are shown. Internal rooting was performed according to the RepC-tree of Petersen et al. (2009). Rhodobacteracean subtrees and the “rhizobial” tree are highlighted by pink and blue boxes, respectively. [file Image2.PDF]

Fig. S3

RepA1A2-Phylogeny

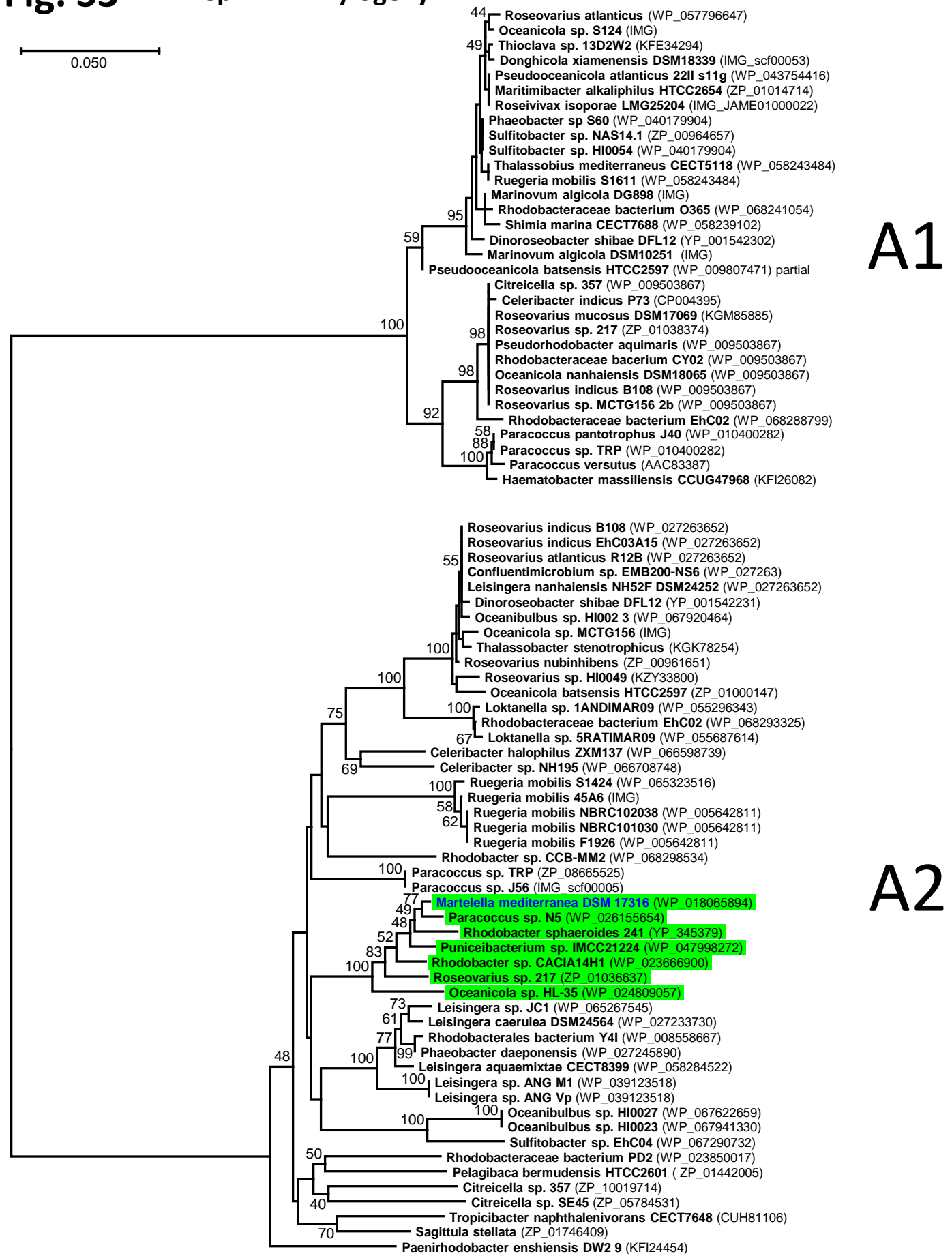

Supplement: Figure S3 — Neighbor Joining tree (p-distances; 100 BR) of RepA partitioning proteins from the RepABC plasmid replication operon of the rhodobacteracean compatibility groups 1 and 2 based on 81 sequences and 386 amino acid positions. Martelella mediterranea DSM 17316T is highlighted in blue. Strains for phylogenetic subanalyses are highlighted in green (Figure 1B, Figure S5). [file Image3.PDF]

Fig. S4

RepB1B2-Phylogeny

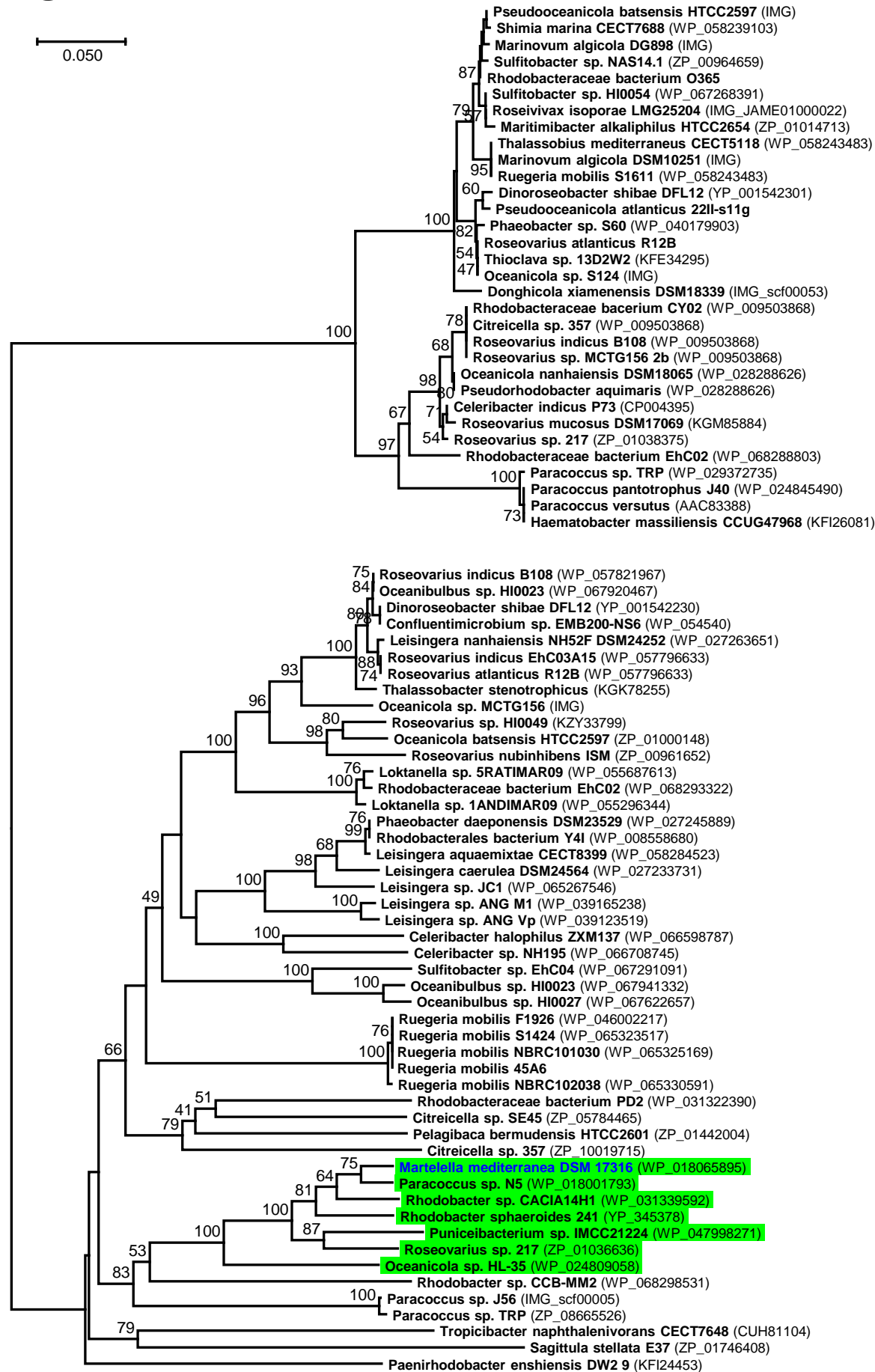

Supplement: Figure S4 — Neighbor Joining tree (p-distances; 100 BR) of RepB partitioning proteins from the RepABC plasmid replication operon of the rhodobacteracean compatibility groups 1 and 2 based on 81 sequences and 225 amino acid positions. [file Image4.PDF]

Fig. S5

RepC1C2-Phylogeny

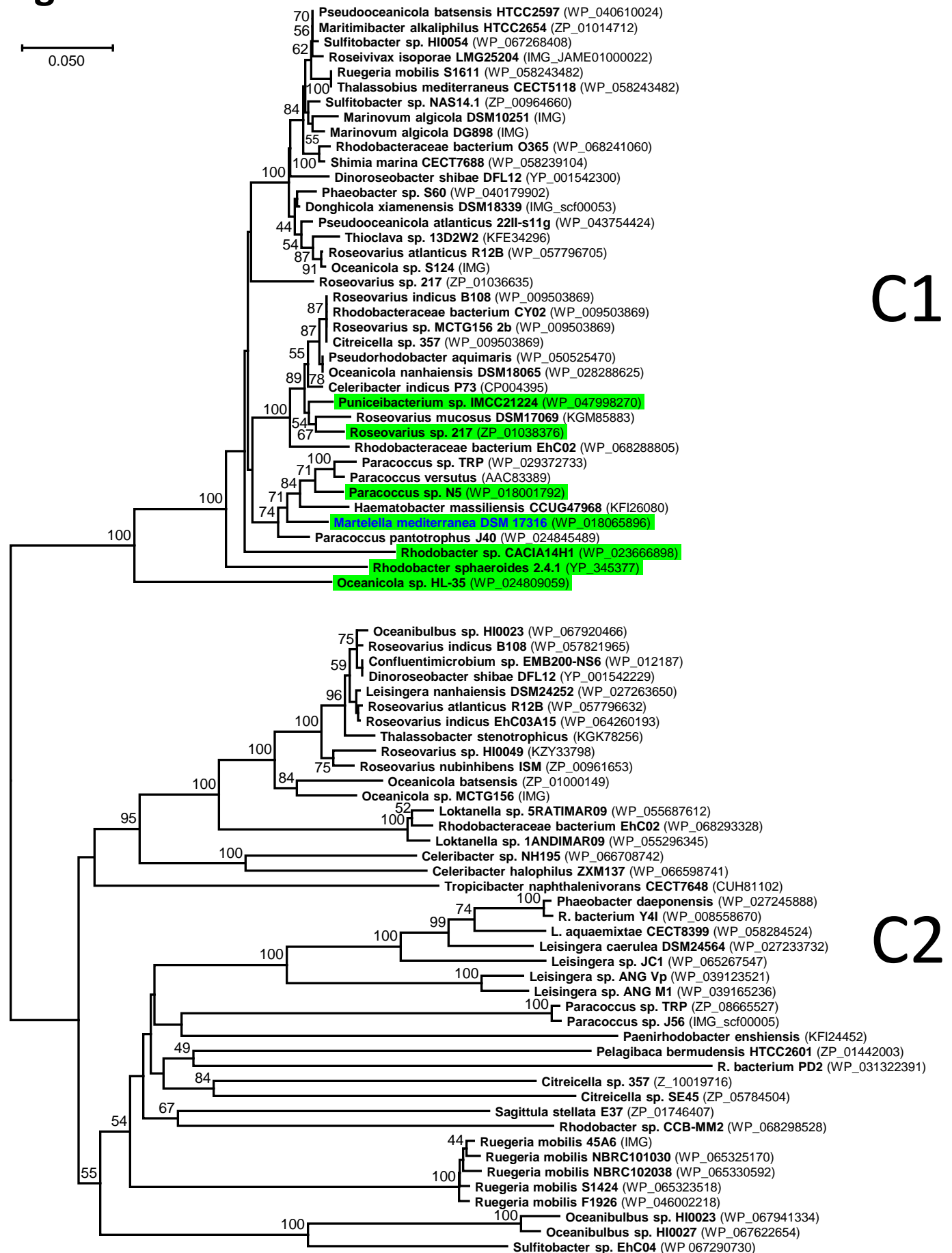

Supplement: Figure S5 — Neighbor Joining tree (p-distances; 100 BR) of RepC replicases from the RepABC plasmid replication operon of the rhodobacteracean compatibility groups 1 and 2 based on 81 sequences and 352 amino acid positions. [file Image5.PDF]

**Fig. S6** (A) RepA2-Phylogeny

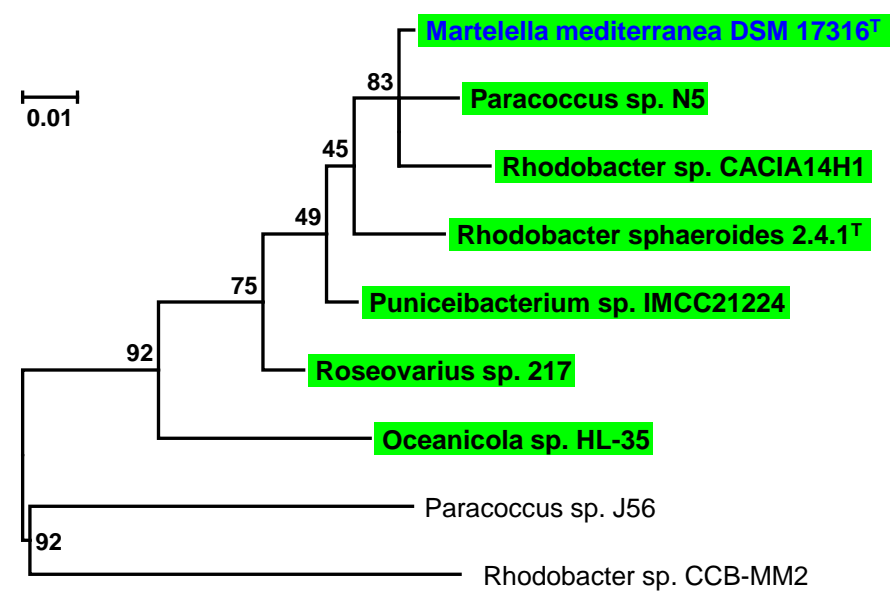

(B) RepB2-Phylogeny

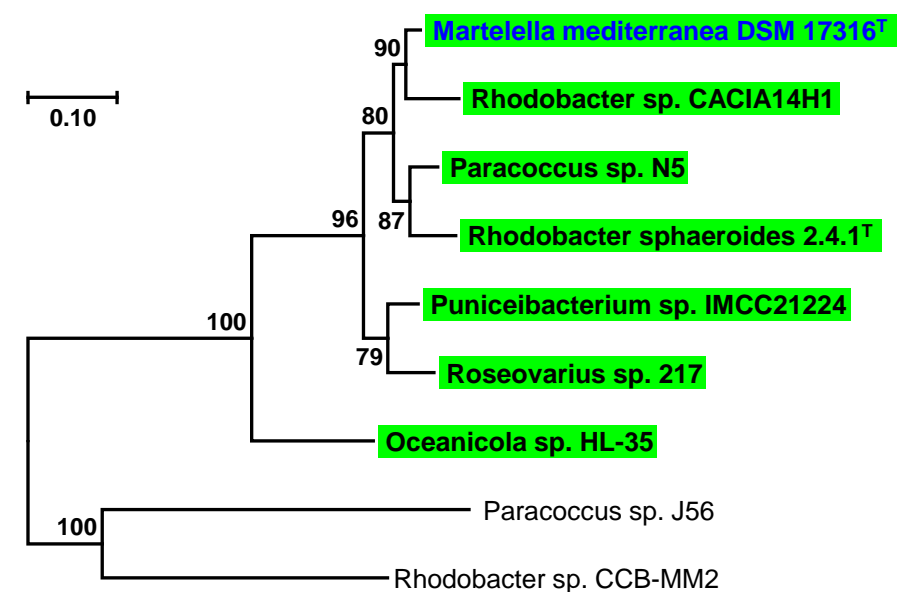

(C) RepA2B2-Phylogeny

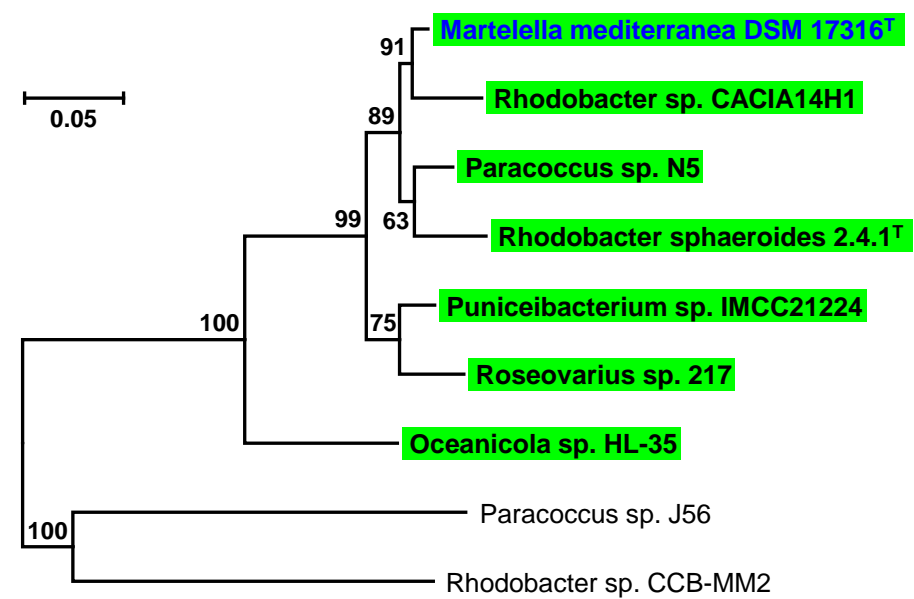

(D) repA2B2-Phylogeny

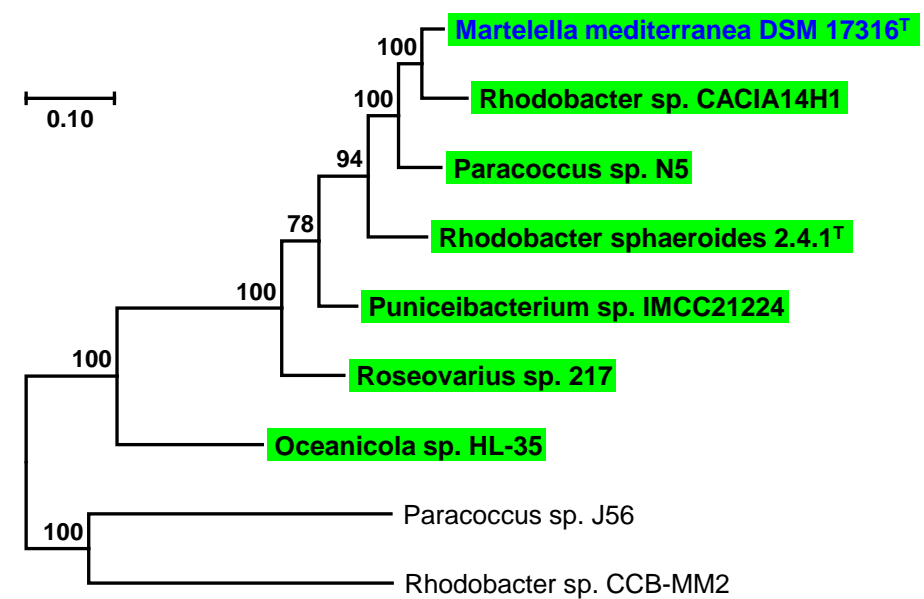

Supplement: Figure S6 — Phylogenetic positioning of the repAB partitioning module from Martelella mediterranea DSM 17316T. Strains with A2B2C1-type plasmid replication systems (Figures S2–S4) are highlighted in bold and green. (A) Maximum Likelihood [ML] tree (RAxML, LG+F+4Γ; 100 BR) of RepA2 proteins based on nine sequences and 394 amino acid positions. (B) ML tree (RAxML, LG+F+4Γ; 100 BR) of RepB2 proteins based on 312 amino acid positions. (C) ML tree (RAxML, LG+F+4Γ; 100 BR) of concatenated RepA2 and RepB2 proteins based on 706 amino acid positions. (D) ML tree (RAxML, GTR+4Γ; 100 BR) of concatenated repA2 and repB2 genes based on 2164 nucleotide positions. [file Image6.PDF]

Fig. S7 (A) RepC1-Phylogeny

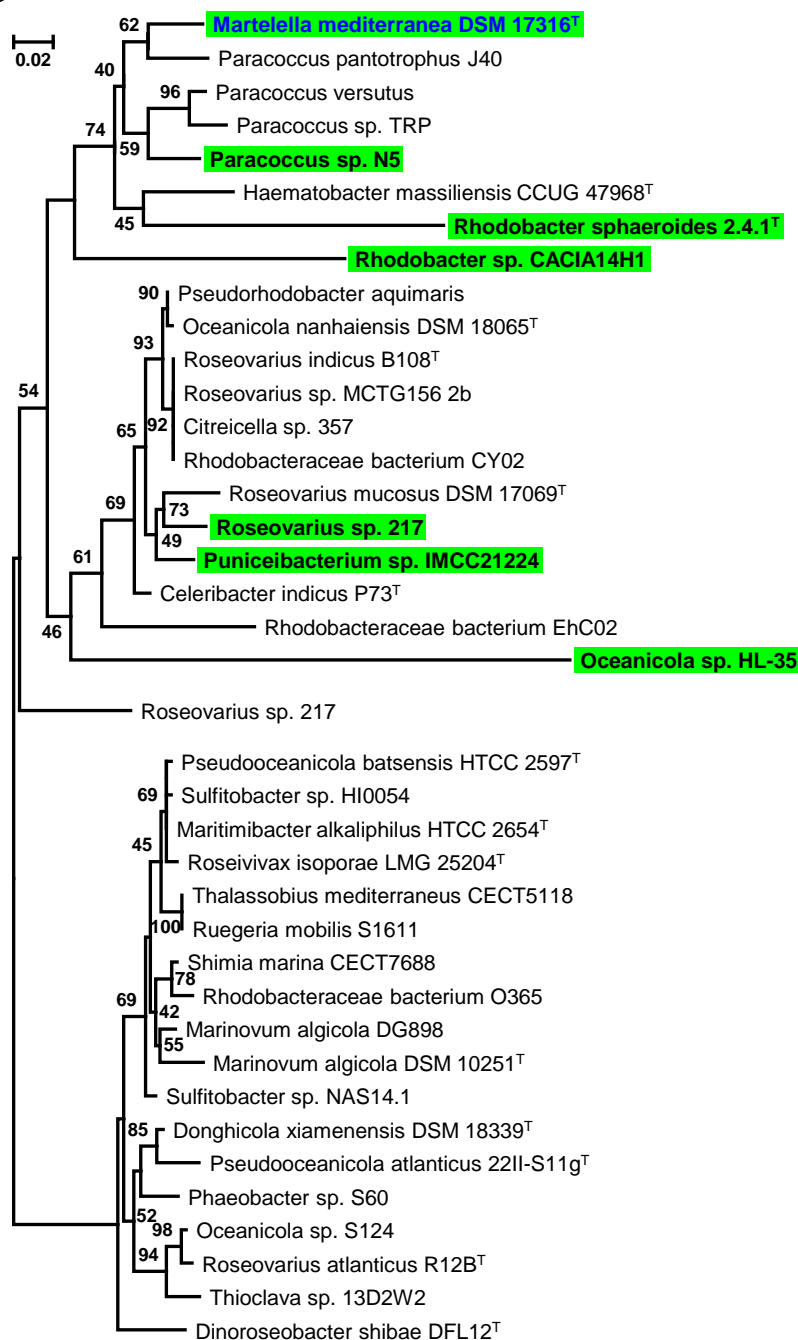

(B) RepC1-Phylogeny

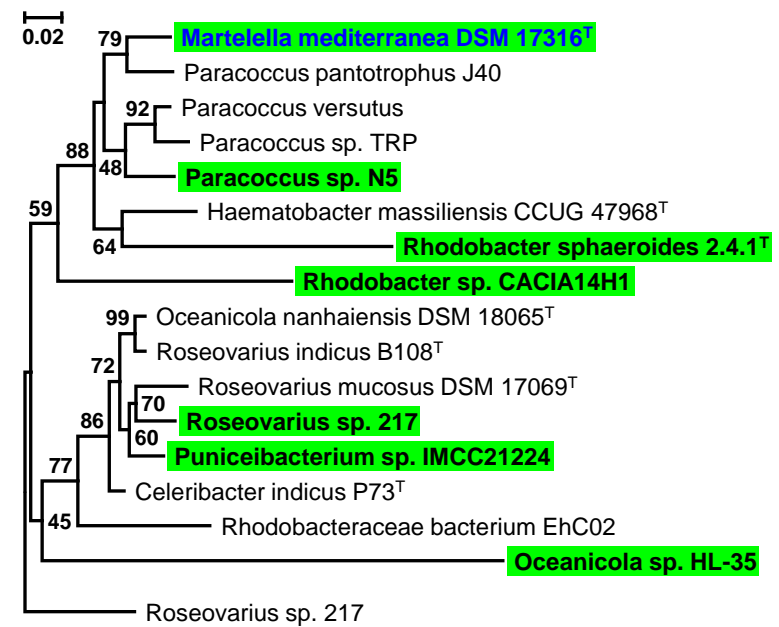

(C) repC1-Phylogeny

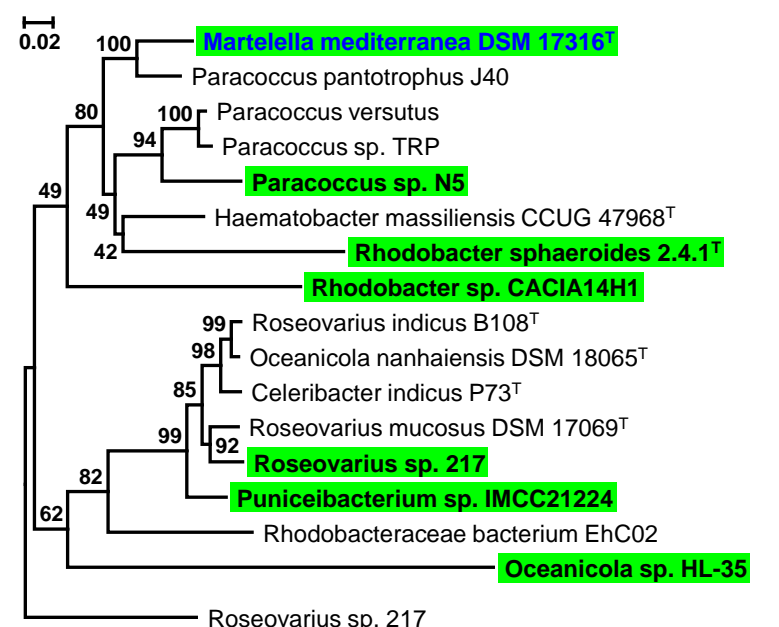

Supplement: Figure S7 — Phylogenetic positioning of the repC replication gene from Martelella mediterranea DSM 17316T. Strains with A2B2C1-type plasmid replication systems (Figures S2–S4) are highlighted in bold and green. (A) Maximum Likelihood tree (ML; RAxML, LG+F+4Γ; 100) of RepC1 proteins based on 39 sequences and 403 amino acid positions. (B) ML tree (RAxML, LG+F+4Γ; 100 BR) of RepC1 proteins based on 17 sequences and 402 amino acid positions. (C) ML tree (RAxML, GTR+4Γ; 100 BR) of repC1 genes based on 17 sequences and 1212 nucleotide positions. [file Image7.PDF]

**Fig. S8** RSCU analysis of *Martelella mediterranea* DSM 17316<sup>T</sup>

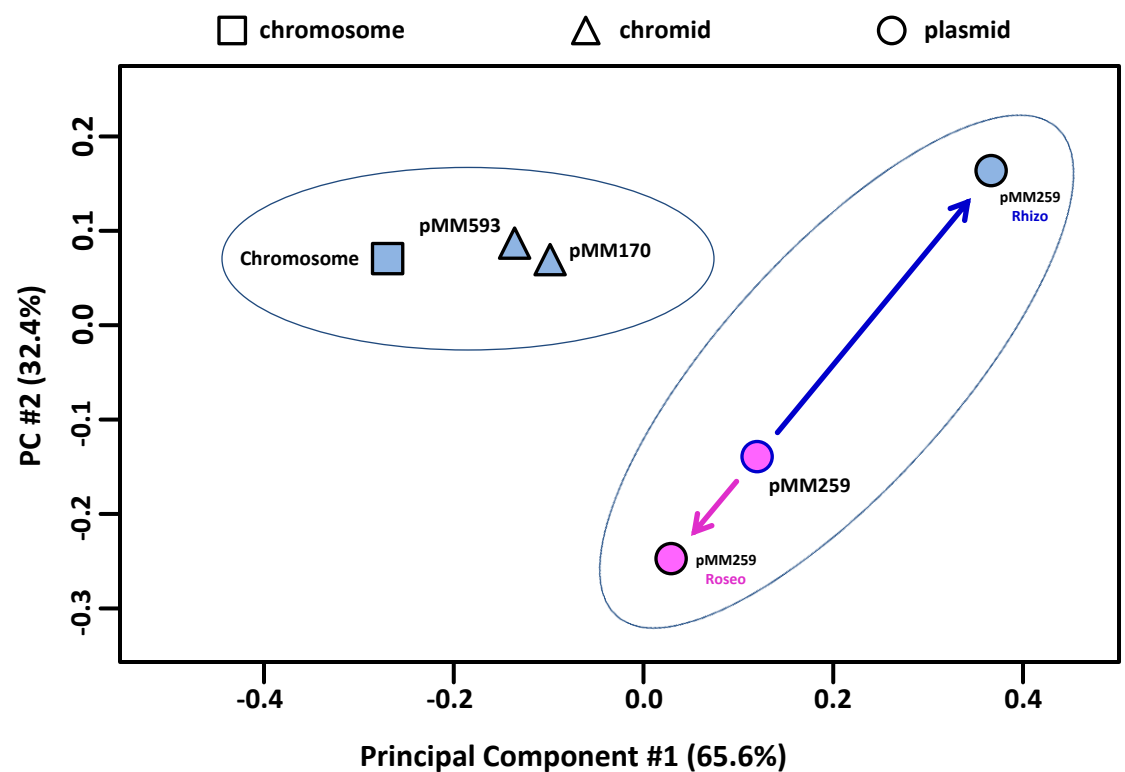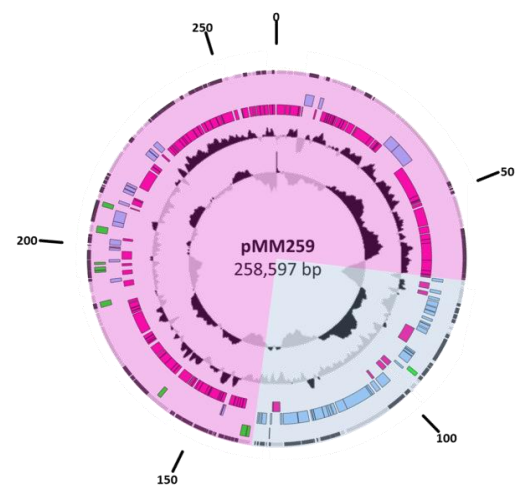

Supplement: Figure S8 — Principal component and cluster analysis of relative synonymous codon usage (RSCU) based on all protein-coding sequences from the four M. mediterranea replicons. The rhodobacteral (Roseo) and rhizobial (Rhizo) specific genes of pMM259 were also analyzed separately. Their distribution is highlighted in the plasmid map in pink or in blue, respectively. Two-dimensional scaling explains 98.0% of the variance. Chromosomes, chromids and plasmids are indicated by squares, triangles and circles, respectively. [file Image8.PDF]
