# Supplementary material for: The Composite 259-kb Plasmid of Martelella mediterranea DSM 17316T–A Natural Replicon with Functional RepABC Modules from Rhodobacteraceae and Rhizobiaceae
Source: Front Microbiol. 2017 Sep 21;8:1787. doi: 10.3389/fmicb.2017.01787 (PMC5613091; doi:10.3389/fmicb.2017.01787)
Supplement: Figure S9 — Plasmid stability tests of the rhizobial and rhodobacteral RepABC-type plasmids pMM259-Roseo, pMM259-Rhizo and pPI88-Roseo in Phaeobacter inhibens DSM 17395 and Agrobacterium tumefaciens C58 (see Figure 4). 3 μl of resuspended bacterial colonies were in parallel spotted on two agar plates with and without the antibiotic kanamycin. [file Image9.PDF]

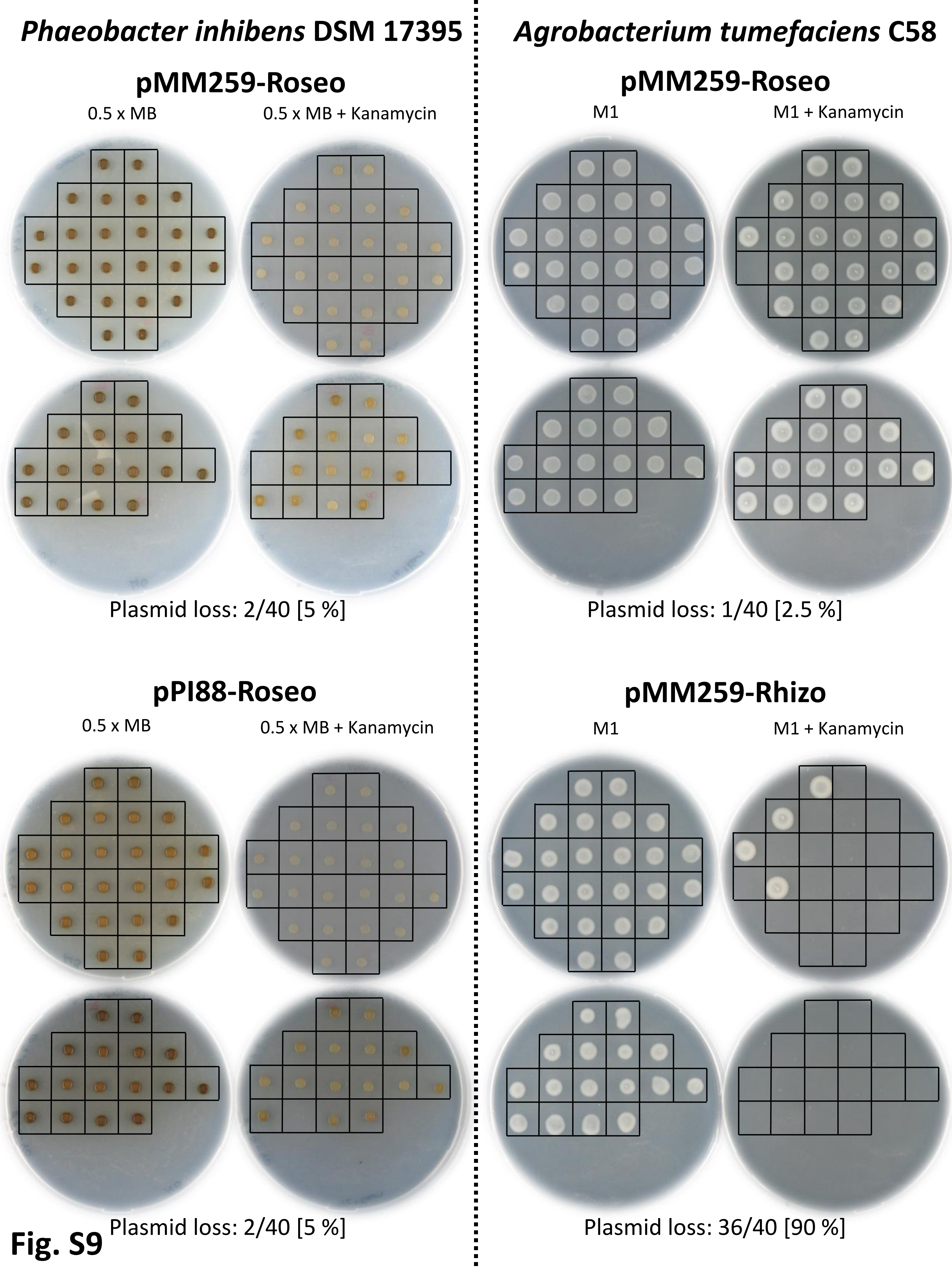

Phaeobacter inhibens DSM 17395
pMM259-Roseo
0.5 x MB
0.5 x MB + Kanamycin
Plasmid loss: 2/40 [5 %]
pPI88-Roseo
0.5 x MB
0.5 x MB + Kanamycin
Plasmid loss: 2/40 [5 %]
Agrobacterium tumefaciens C58
pMM259-Roseo
M1
M1 + Kanamycin
Plasmid loss: 1/40 [2.5 %]
pMM259-Rhizo
M1
M1 + Kanamycin
Plasmid loss: 36/40 [90 %]

Fig. S9
