# Supplementary material for: The Composite 259-kb Plasmid of Martelella mediterranea DSM 17316T–A Natural Replicon with Functional RepABC Modules from Rhodobacteraceae and Rhizobiaceae
Source: Front Microbiol. 2017 Sep 21;8:1787. doi: 10.3389/fmicb.2017.01787 (PMC5613091; doi:10.3389/fmicb.2017.01787)
Supplement: Supplementary file 14 [file DataSheet1.DOCX]

**Supplemental Material S1**

*Phylogenetic subanalyses of RepA-2, RepB-2 and RepC-1 sequences*

Our outside-in approach allowed determining the evolutionary origin of the xenologous RepABC plasmid module (A2B2C1), but the question regarding its closest relative remained so far unanswered. RepA2 und RepB2 phylogenies showed that *M. mediterranea* is located in a distinct subtree (green color; 100% BP; Figs. S3, S4), whereas the corresponding replicases are clearly not monophyletic and distributed over the RepC1 subtree (Fig. S5). However, the sequence sampling for Figures S3, S4 and S5 was based on a comprehensive selection of RepABC modules from compatibility groups -1 and -2 and the presence of two highly distinct subtrees in each phylogeny might bother their topologies via LBA effects. Accordingly, we focused our subanalyses on the seven strains with an A2B2C1-type operon highlighted in green. The RepA2 subtree revealed a close relationship between *M. mediterranea*, *Paracoccus* sp. N5 and *Rhodobacter* sp. CACIA14H1 (83% BP; Fig. S6A), and subtree RepB2 moreover showed a specific association between *Martelella* and the strain CACIA14H1 (90% BP; Figs. S6B). *Rhodobacter sphaeroides* 2.4.1^T^ is associated with the three sequences to an exclusion of *Puniceibacterium* sp. IMCC21224 and *Roseovarius* sp. 217, but its actual position remains unclear even in the RepA2B2 tree based on a concatenated protein alignment (Fig. S6C). Accordingly, and in order to increase the resolution among the closely related sequences, we calculated a final *repA2B2* phylogeny based on concatenated nucleotide sequences (Fig. 1B, Fig. S6D). This well resolved tree clearly confirms the sistergroup relationship between *Rhodobacter* sp. CACIA14H1 and *Martelella* (100% BP), followed by serial branching *Paracoccus* sp. N5 and *R. sphaeroides* 2.4.1^T^ sequences (100% BP, 94% BP).

The scattered distribution of the ‘green’ replicases from A2B2C1 modules in the phylogenetic subtree C1 (Fig. S5) is exemplified by the nested positioning of *M. mediterranea* between *Haematobacter massiliensis* CCUG 47968^T^ and *Paracoccus pantotrophus* J40 both harboring archetypal A1B1C1 modules (Table 1). The resolution of the topology could be improved by a limitation to RepC1 sequences and the tree shows very long branches for *R. sphaeroides* 2.4.1^T^, *Rhodobacter* sp. CACIA14H1 and *Oceanicola* sp. HL-35 (Fig. S7A). Accordingly, our set of phylogenetic RepC analyses documented that their basal positioning in Figure S5 is a typical LBA artifact generated by divergent RepC2 sequences (Fig. S2, Fig. S6, Fig. S7A; Philippe et al., 2005). A further reduction of the dataset focused on the closest relatives of the ‘green’ RepC1 sequences resulted in an unchanged topology with a higher statistical support (compare Figs. S6A, B), but the probably best resolution was again obtained for the *repC1* tree based on nucleotide sequences (Fig. 1C, Fig. S7C). This analysis validated the scattered distribution of the ‘green’ *repC1* sequences supported by considerable bootstrap values, and it moreover showed a specific relationship of the *repC1* of *M. mediterranea* and *P. pantotrophus* (100% BP) to an exclusion of *H. massiliensis*, *R. sphaeroides* 2.4.1^T^ and three further *Paracoccus* strains. The common origin of these strains to an exclusion of *Rhodobacter* sp. CACIA14H1 is solidly supported by our protein and nucleotide analyses (88% BS [Fig. S7B], 80% BS [Fig. S7C]). The branching pattern of the ‘green’ replicases is contradictory to the presumed concerted evolution of the whole A2B2C1 operon (Table 1), thus indicating that *repC1* genes were replaced several times by closely related equivalents. In conclusion, the phylogenetic analyses allowed us to pinpoint the evolutionary origin of the RepA2B2C1 plasmid replication system from *M. mediterranea*. Its contradictory affiliation with *Rhodobacter* sp. CACIA14H1 (*repA2B2*; 100% BP, Fig. 1B, Fig. S6D) and *P. pantotrophus* J40 (*repC1*; 100% BP, Fig. 1C, Fig. S7C) clearly documents that the genuine rhodobacteracean donor of *Martelella*´s A2B2C1 module – grouping as closest relative in all three phylogenies - has not been detected yet.

**Reference**

Philippe, H., Lartillot, N., and Brinkmann, H. (2005). Multigene analyses of bilaterian animals corroborate the monophyly of Ecdysozoa, Lophotrochozoa, and Protostomia. *Mol. Biol. Evol.* 22, 1246–1253. doi:10.1093/molbev/msi111.
